# Excited-State Properties and Relaxation Pathways of Selenium-Substituted Guanine Nucleobase in Aqueous Solution and DNA Duplex

**DOI:** 10.1021/acs.jpcb.0c10855

**Published:** 2021-02-11

**Authors:** Ye-Guang Fang, Danillo Valverde, Sebastian Mai, Sylvio Canuto, Antonio Carlos Borin, Ganglong Cui, Leticia González

**Affiliations:** † Key Laboratory of Theoretical and Computational Photochemistry, Ministry of Education, College of Chemistry, Beijing Normal University, Beijing 100875, P. R. China; ‡ Institute of Physics, University of São Paulo, Rua do Matão 1371, São Paulo, SP 05508-090, Brazil; § Photonics Institute, Vienna University of Technology, Gußhausstraße 27-29, 1040 Vienna, Austria; ∥ Institute of Theoretical Chemistry, Faculty of Chemistry, University of Vienna, Währinger Straße 17, 1090 Vienna, Austria; ⊥ Department of Fundamental Chemistry, Institute of Chemistry, University of São Paulo, Av. Prof. Lineu Prestes 748, 05508-000. São Paulo, SP Brazil

## Abstract

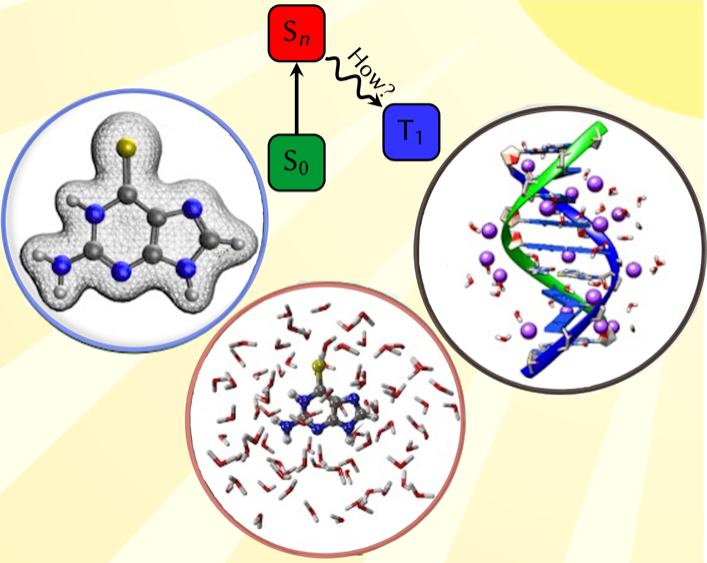

The excited-state properties and relaxation mechanisms after light irradiation of 6-selenoguanine (6SeG) in water and in DNA have been investigated using a quantum mechanics/molecular mechanics (QM/MM) approach with the multistate complete active space second-order perturbation theory (MS-CASPT2) method. In both environments, the S_1_^1^(n_Se_π_5_^*^) and S_2_^1^(π_Se_π_5_^*^) states are predicted to be the spectroscopically dark and bright states, respectively. Two triplet states, T_1_^3^(π_Se_π_5_^*^) and T_2_^3^(n_Se_π_5_^*^), are found energetically below the S_2_ state. Extending the QM region to include the 6SeG-Cyt base pair slightly stabilizes the S_2_ state and destabilizes the S_1_, due to hydrogen-bonding interactions, but it does not affect the order of the states. The optimized minima, conical intersections, and singlet–triplet crossings are very similar in water and in DNA, so that the same general mechanism is found. Additionally, for each excited state geometry optimization in DNA, three kind of structures (“up”, “down”, and “central”) are optimized which differ from each other by the orientation of the C=Se group with respect to the surrounding guanine and thymine nucleobases. After irradiation to the S_2_ state, 6SeG evolves to the S_2_ minimum, near to a S_2_/S_1_ conical intersection that allows for internal conversion to the S_1_ state. Linear interpolation in internal coordinates indicate that the “central” orientation is less favorable since extra energy is needed to surmount the high barrier in order to reach the S_2_/S_1_ conical intersection. From the S_1_ state, 6SeG can further decay to the T_1_^3^(π_Se_π_5_^*^) state via intersystem crossing, where it will be trapped due to the existence of a sizable energy barrier between the T_1_ minimum and the T_1_/S_0_ crossing point. Although this general S_2_ → T_1_ mechanism takes place in both media, the presence of DNA induces a steeper S_2_ potential energy surface, that it is expected to accelerate the S_2_ → S_1_ internal conversion.

## 1 Introduction

In the prebiotic age, the protection of the ozone layer was not efficient and the flux of UV radiation on Earth was much higher than it is nowadays, resulting in an hostile atmosphere capable of causing severe damage to DNA.^1^ In these conditions, canonical purine (adenine and guanine) and pyrimidine (cytosine, thymine, and uracil) nucleobases emerged as very photostable compounds, able to preserve the genetic code from the deleterious effects of UV radiation. Photostability originates from an efficient excited-state decay that releases the excess of energy via ultrafast and efficient radiationless deactivation processes.^2−5^ Today, it is well established that low-energy conical intersections that allow the system to return to the electronic ground state in a short time scale are responsible for the excited-state decay efficiency.^6−11^

Small chemical modifications can drastically alter the inherent photostability of nucleobases.^12^ Particular attention has been devoted to thiobases analogues—in which oxygen atoms are replaced by sulfur—because, unlike their canonical counterparts, intersystem crossing results in high quantum yields of triplet states.^13−20^ For instance, 6-thioguanine (6tG) has an intersystem crossing quantum yield of ca. 60%.^17,21^ The radical nature of the triplet states render thiobases potential applications as photosensitizers for photodynamic therapy against a variety of ailments.^22^ Examples are 2,4-dithiothymine,^23^ 6-thio-2-deoxyguanine,^24^ or 2,6-dithiopurine.^25^ A natural extension in this direction are seleno-nucleobases, where selenium replaces oxygen or sulfur. Current studies^26−29^ point out that Se-nucleobases can form stable RNA,^30^ DNA duplex,^31^ and G-quadruplex structures.^32^ They exhibit advantageous red-shift absorption spectra in comparison with their thio-counterparts,^33,34^ and most importantly, due to their heavier atom, a faster intersystem crossing has been observed compared to their thio-analogs—albeit with a shorter triplet state lifetime.^34^

Theoretical theory is particularly well suited to unravel the photophysical mechanisms responsible for the efficient electronic population of excited states.^35^ However, all previous studies in sulfur- and selenium-substituted nucleobases^36−43^ have systematically excluded the effect of the DNA environment due to the complexity involved, despite it could strongly influence the photophysical properties of its chromophores.^44^

In this paper, we employ hybrid Quantum Mechanics/Molecular Mechanics (QM/MM) techniques^45,46^ to achieve a realistic description of the biomolecular environment by including the effect of solvation and that of the double helix DNA background on 6-selenoguanine (6SeG). According to previous theoretical studies in gas phase,^47^ in 6SeG the S_2_^1^(ππ*) state transfers its population to the S_1_^1^(nπ*) state via a conical intersection, from which the triplet states are formed. More recently, three selenium-substituted uracils (2SeU, 4SeU, and 2,4SeU) have also been investigated in gas phase and similar excited-state relaxation pathways to triplet states are reported.^48,49^ The primary goal of this contribution is to examine whether the main photophysical events of 6SeG are affected by the presence of water and/or biological media.

## 2 Computational Details

### 2.1 System Setup

**Figure 1:**
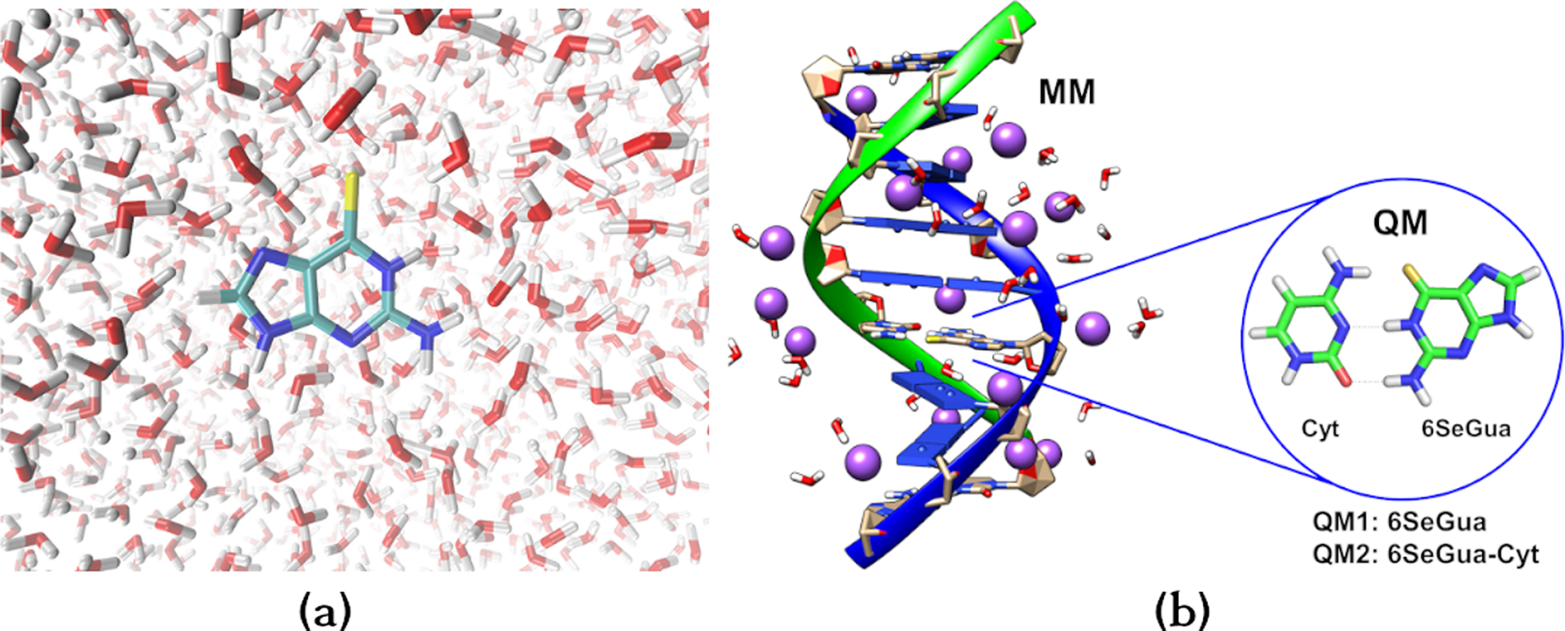
QM/MM system setup. (a) 6SeG in water solution. (b) 6SeG embedded in DNA environment. Two different QM regions are indicated: only 6SeG (QM1) or 6SeG paired with cytosine (QM2). All remaining atoms are included in the MM subsystem.

The 6SeG molecule was studied both in water solution and in a DNA environment. In both cases, first classical molecular dynamics (MD) simulations were performed with the Amber16 package^50^ in order to obtain initial structures to use in subsequent QM/MM calculations. Intra- and intermolecular parameters were extracted from the generalized Amber force field (GAFF),^51^ while the force field parameters of the selenium atom were taken from the parametrization of the 2-selenouridine molecule embedded in aqueous solution.^52^ The RESP (Restrained Electrostatic Potential) procedure^53^ was performed to get the set of atomic charges at the B3LYP/cc-pVDZ level.^54−58^ In the water simulations, the TIP3P model^59^ was used with a system comprising one solute molecule surrounded by 5406 water molecules inside of a truncated octahedron simulation box of 12 Å (Figure 1a). As no constraint was applied during the simulation, a reduced time step of 0.5 fs was employed. Initially, the system was minimized with the steepest descent algorithm, followed by heating in the *NVT* ensemble for a total time of 100 ps, scaling the temperature from 0 to 300 K. Afterward, an equilibration regime was applied for 1 ns in the *NPT* ensemble in normal conditions of temperature and pressure (*P* = 1 bar and *T* = 300 K). The final production was carried out for 10 ns, from which the last snapshot was employed to investigate the photophysics of 6SeG in water with a QM/MM approach.

In order to include the DNA environment, a duplex with the sequence 5′-ATGGTGCAC-3′ and 3′-TACCACGTG-5′ was employed (Figure 1b). This sequence was first constructed by using a nucleic acid builder tool in Amber16 package as in previous experimental studies.^60^ The DNA duplex system was solvated in a water box of 56 × 59 × 69 Å. DNA, waters, and counterions were described with default ff99SB force fields^61^ and TIP3P model.^59^ Then, 1000 MM minimization cycles with frozen DNA were carried out, followed by 2000 cycles without any geometric constraints. The minimized system was heated to 300 K in a 40 ps equilibration MD simulation, followed by 10 ns MD simulations. As in water solution, for the QM/MM study the final MD snapshot was used, in which the oxygen atom of the fourth G base was replaced with a selenium atom (5′-ATG-SeG-TGCAC-3′:3′-TAC-C-ACGTG-5′).

### 2.2 QM/MM Calculations

The QM/MM calculations were performed using either MOLCAS8.0^62,63^ or OpenMOLCAS^64,65^ interfaced with TINKER6.3.^66^ In aqueous solution, the QM region contained the 6SeG molecule while all the water molecules were described with MM. In DNA, two different QM regions were constructed to explore effects of interbase hydrogen-bonding interaction, see Figure 1b. The first (QM1) includes only 6SeG while the second contains the 6SeG paired with a cytosine base (6SeG-Cyt). In both models, all remaining atoms from the DNA duplex, sugar, phosphates, water molecules, and counterions are left in the MM region.

**Figure 2:**
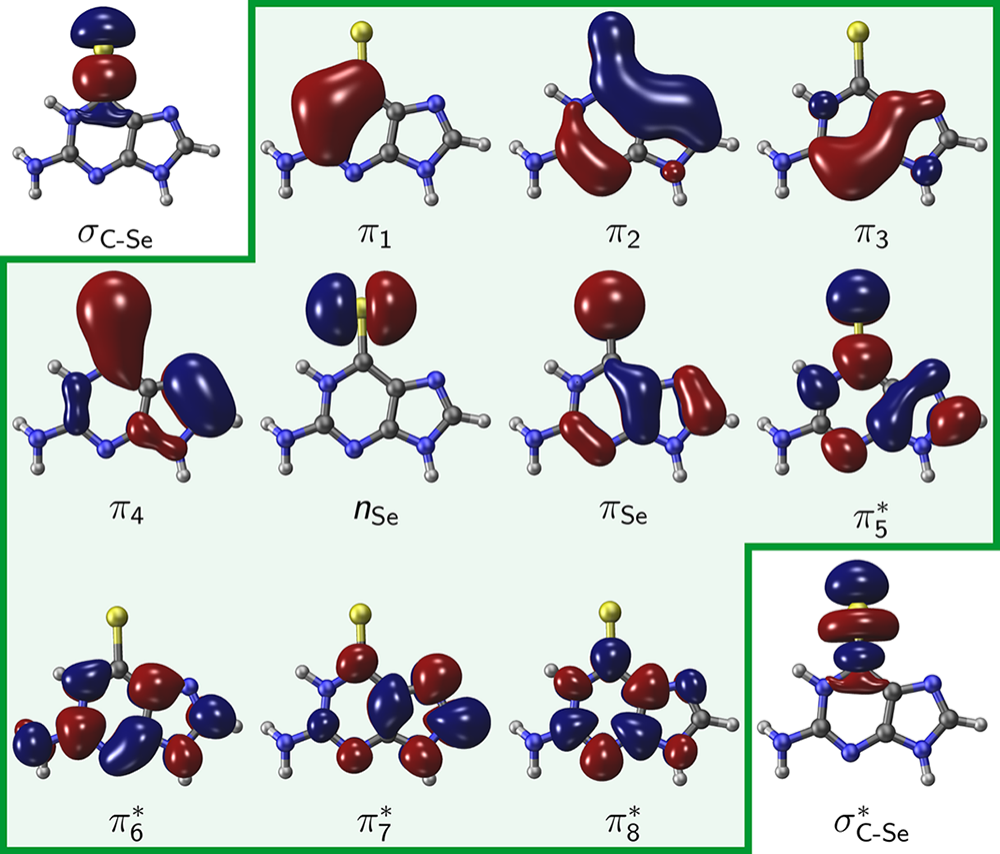
Orbitals included in the active spaces used for the QM/MM calculations in water. In green are shown the orbitals of the (12,10) active space used in the optimizations. The two additional σ and σ* orbitals located on the C=Se moiety are only included to refine single point energies.

Regardless of the environment (only water or solvated DNA) the QM region was described with the state-averaged complete active space self-consistent field (SA-CASSCF) method^67^ for geometry optimizations and the multistate second-order perturbation theory (MS-CASPT2) approach^68,69^ on top to refine single-point energies. A total of 4 singlets and 3 triplets were included in the state-average procedure. During the SA-CASSCF calculations, C, N, O, and H atoms were described with cc-pVDZ and the Se atom with the aug-cc-pVDZ basis set.^58,70^ The active space consisted of 12 electrons distributed over the 10 orbitals; see Figure 2 for the active orbitals in water and Figures S1 and S2 for the orbitals within the DNA environment. The MS-CASPT2 energy calculations were carried out employing larger atomic basis sets, specifically cc-pVTZ for C, N, O and H, and aug-cc-pVTZ for Se. In this case, the active space was augmented to 14 electrons in 12 orbitals with σ and σ* orbitals located on the C=Se moiety; see Figure 2.

An imaginary level shift of 0.2 au was employed to avoid the issues of intruder states.^71^ The IPEA shift value was set to zero.^72,73^ The Cholesky decomposition technique for two-electron integrals was employed to accelerate CASSCF and MS-CASP2 calculations.^74^ Spin–orbit couplings were obtained at the MS-CASPT2 level within the atomic mean-field (AMFI) approximation.^75−77^ The effective spin–orbit couplings are expressed as follows:
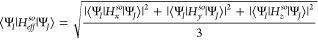
in which Ψ_*I*_ and Ψ_*J*_ are the perturbatively modified electronic wave functions of the corresponding singlet and triplet states; *H*_*x*_^*so*^, *H*_*y*_^*so*^, and *H*_*z*_^*so*^ are the *x*, *y*, and *z* components of the spin–orbit operator.

We would like to emphasize that only adiabatic states are considered throughout this work. In this state representation, population transfers among states of the same multiplicity are induced by nonadiabatic couplings, which become extremely large close to conical intersections. Population transfer between different multiplicities is mediated by spin–orbit couplings, whose magnitude is independent of the energy gaps. Hence, in addition to the location of relevant minimum-energy singlet–triplet crossings, we also computed the spin–orbit coupling matrix elements at these crossings.

For comparison, in addition to the QM/MM calculations of 6SeG in the presence of explicit water molecules, we also carried out computations with the polarizable continuum model (PCM) that considers solvent effects only implicitly.^78,79^

To save computational effort, only the last saved snapshot from the MD simulations was used as a starting point for the optimization of excited state geometries with QM/MM. However, in order to verify whether this approach suffices to predict deactivation pathways, we also performed single point vertical excitation energy calculations considering ten randomly selected snapshots taken from the classical simulation in both environments (see Tables S1–S3). The superposition of the selected snapshots shows that the nearest water molecules are well distributed around the 6SeG (see Figure S3), which means that the selected snapshots are representative and give a good representation of the solvent configurations. Since the calculated root-mean-square deviation is small for the two lowest singlet states, with the largest deviation of 0.07 eV—within the accepted error of the method—we conclude that the use of one snapshot for the following discussion of the relaxation pathways is reasonable.

## 3 Results and Discussion

### 3.1 Equilibrium Structures

**Figure 3:**
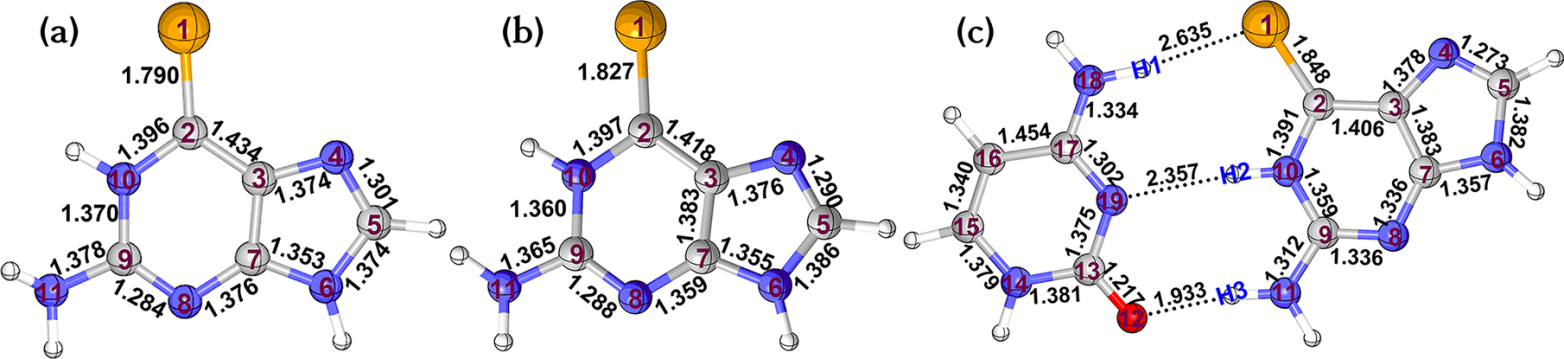
QM(CASSCF)/MM optimized structures of 6SeG in the electronic ground state, in aqueous solution (a), in DNA (b), and in a pair with cytosine (c). Selected bond lengths are in Å, and atomic numbering is given.

Figure 3 shows the optimized geometries of 6SeG in the electronic ground state (S_0_) in water solution (a) and in DNA (b), as obtained with the QM(CASSCF)/MM approach. In aqueous solution, 6SeG has a structure nearly planar with the two hydrogen atoms of the amino group out of the six-membered ring plane by ca. 26°. The C=Se bond length is computed to be 1.790 Å, slightly shorter than that predicted in gas phase,^47^ 1.821 Å.

The S_0_ structure of 6SeG embedded in DNA (Figure 3b) and that within the explicit 6SeG-Cyt base pair (Figure 3c) are also essentially planar. Noticeable is that the C=Se bond lengths are 1.827 and 1.848 Å, respectively, much longer than the corresponding C=S bond length observed in thio-counterpart (∼1.650 Å).^16,38,80^ As expected, the explicit consideration of three hydrogen bonds in 6SeG-Cyt influence the geometric parameters of the 6SeG moiety. Accordingly, the hydrogen-bonding interaction related to the Se and N10 atoms is much weaker than that related to the N11 atom, as suggested by their corresponding bond lengths. The two hydrogen bonds, Se···H20 and N19···H21, are 2.635 and 2.357 Å, whereas the O12···H22 bond length is 1.933 Å. Since the former two hydrogen bonds are much weaker, there are small effects on the geometric parameters around the C2–Se1 moiety. Likewise, the stronger O12···H3 hydrogen-bonding interaction affects the nearby bond lengths; for example, the C9–N11 and N8–C9 bond lengths are 1.365 and 1.288 Å in 6SeG, but 1.312 and 1.336 Å in 6SeG-Cyt.

Overall, the S_0_ structures of 6SeG in both water and DNA environments are similar except for the C9–N11 and C=Se bond lengths–likely due to the pairing cytosine.

### 3.2 Spectroscopic Properties

Table 1 collects the vertical excitation energies of 6SeG computed in water solution and within DNA, without and with the explicit consideration of the paired cytosine (QM1 vs QM2). We shall first discuss the results in water solution. The first singlet excited state, S_1_^1^(n_Se_π_5_^*^), corresponds to a dark state predicted at 3.13 eV in implicit solution and 2.62 eV with explicit solvent. Relative to the S_0_ state, this state is due to a single electronic excitation from the n_Se_ nonbonding orbital localized on the Se atom, to the π_5_^*^ antibonding orbital. As mentioned above, there is a difference of around 0.5 eV between the results obtained with the two solvation models. This difference is expected since the degrees of freedom of the solvent are neglected in the implicit solvation model. As known in the literature,^82^ lone pairs states are more affected by the explicit solvation, especially in polar solvent, due to the formation of solute–solvent hydrogen bonds.

**Table 1:** Vertical Excitation Energies (*ΔE*, eV) and Related Oscillator Strengths (*f*) of 6SeG in Water Computed at the MS-CASPT2/PCM and MS-CASPT2(14,12)/MM Levels of Theory and in DNA with QM1(MS-CASPT2(14,12))/MM and QM2(MS-CASPT2(14,12))/MM (in Parentheses)

	water				DNA	
	MS-CASPT2/PCM^a^		QM(MS-CASPT2)/MM^b^		QM(MS-CASPT2)/MM^c^	
states	*ΔE*	*f*	*ΔE*	*f*	*ΔE*	*f*
S_1_ ^1^(n_Se_π_5_^*^)	3.13	0.000	2.62	0.000	2.56 (2.79)	0.000 (0.006)
S_2_ ^1^(π_Se_π_5_^*^)	3.29	0.577	3.51	0.570	3.40 (3.29)	0.525 (0.517)
S_3_ ^1^π_Se_π_6_^*^	4.73	0.006	4.43	0.016		
T_1_ ^3^(π_Se_π_5_^*^)	2.60		2.55		2.35 (2.48)	
T_2_ ^3^(n_Se_π_5_^*^)	2.88		2.59		2.51 (2.80)	

a Structure optimized with MS-CASPT2(12,10).

b Structure in water optimized with QM(CASSCF(12,10))/MM.

c Structures in DNA optimized with QM(CASSCF(12,10))/MM: 6SeG (6SeG-Cyt).

The S_2_^1^(π_Se_π_5_^*^) state is computed at 3.29 eV in implicit water and at 3.51 eV with explicit solvent. This is a singly excited configuration from the π_Se_ orbital localized on the Se atom to the π_5_^*^ orbital, and it is the bright state (*f* = 0.577). It is encouraging that our calculation with QM(MS-CASPT2)/MM level is in excellent agreement with experimental data that reported an absorption maximum of 6SeG in aqueous buffer solution at 357 nm (3.47 eV).^34^ Thus, this electronic transition is responsible of the first peak of the electronic absorption spectrum of 6SeG in water (see Figure S4).

The third singlet excited state, S_3_^1^(π_Se_π_6_^*^), is around 0.9 eV higher in energy above the S_2_^1^(π_Se_π_5_^*^) state at the QM(MS-CASPT2)/MM level of theory. The corresponding oscillator strength is 0.016—about 1 order of magnitude lower than that observed for the electronic transition to the S_2_^1^(π_Se_π_5_^*^) state, meaning that its contribution to the first absorption band will be small. Accordingly, it will not be considered hereafter.

We have also calculated the two lowest-lying triplet excited states. They have the same electronic transition characters as the singlet counterparts S_1_^1^(n_Se_π_5_^*^) and S_2_^1^(π_Se_π_5_^*^), i.e., T_1_^3^(π_Se_π_5_^*^) and T_2_^3^(n_Se_π_5_^*^). Their vertical excitation energies at the QM(MS-CASPT2)/MM level of theory are 2.55 and 2.59 eV, respectively, and are overestimated with the PCM model, particularly the T_2_. The next triplet state, which is described by a T_3_^3^(π_Se_π_6_^*^) transition, is situated around 1 eV above the S_2_^1^(π_Se_π_5_^*^) state; thus, they are not expected to be accessed from the bright state and are not considered further.

We now proceed to discuss the excitation energies of 6SeG in DNA. As can be seen, both QM1 and QM2 regions (i.e., 6SeG and 6SeG-Cyt) predict the same energetic order of the excited singlet and triplet states and wave function character as in solution. The S_1_ state is spectroscopically dark with nπ* character, and the S_2_ state is bright due to the ππ* character. In the S_1_^1^(n_Se_π_5_^*^) state the π_5_^*^ orbital is delocalized all over including the C=Se double bond. In the S_2_^1^(π_Se_π_5_^*^) state, the π_5_^*^ orbital is the same as that in the S_1_^1^(n_Se_π_5_^*^) state, while the π_Se_ orbital is mainly composed of the p_z orbital of Se with some minor contribution from the C3–C7 bond in either 6SeG or 6SeG-Cyt in DNA. It is worth mentioning that the lowest-energy states of 6SeG-Cyt are basically described by local excited states on 6SeGua and Cyt, respectively, due to the high energy difference between the excited states computed for each of them separately, which are below about 3 eV for 6SeG and 5 eV for Cyt. This is confirmed by QM(MS-CASPT2)/MM calculated vertical excitation energies on 6SeG-Cyt: the S_3_ (4.64 eV) and S_4_ (4.77 eV) states are local excitations on cytosine and charge transfer states, respectively, which are on average 1.4 eV higher than S_2_ (see Table S6). Therefore, charge transfer states are placed at higher energetic regions and the state character of the 6SeG-Cyt S_1_ and S_2_ lowest states are preserved in relation to those observed for the QM1 scheme.

Curiously, the hydrogen-bonding interaction that appears when the cytosine is treated quantum mechanically does not change the characters of the S_1_ and S_2_ states as aforementioned but it considerably affects their energies. The QM1(MS-CASPT2)/MM vertical excitation energies of S_1_ and S_2_ states are predicted to be 2.56 and 3.40 eV and are increased and decreased to 2.79 and 3.29 eV, respectively, with QM2(MS-CASPT2)/MM. This means that the hydrogen-bonding interaction stabilizes the S_2_^1^(π_Se_π_5_^*^) state by 0.11 eV but destabilizes the S_1_^1^(n_Se_π_5_^*^) state by 0.23 eV. A similar situation is found in T_1_^3^(π_Se_π_5_^*^) and T_2_^3^(n_Se_π_5_^*^) states. Their vertical excitation energies are predicted as 2.35 and 2.51 eV with QM1 but 2.48 and 2.80 eV with QM2, respectively.

It is apparent that the QM(MS-CASPT2)/MM approach predicts similar vertical excitation energies regardless of water or DNA environment, except for the fact that the hydrogen-bonding interactions destabilizes the S_1_ and T_2_ states by 0.23 and 0.29 eV at the QM(MS-CASPT2)/MM level.

### 3.3 Excited-State Minima

**Figure 4:**
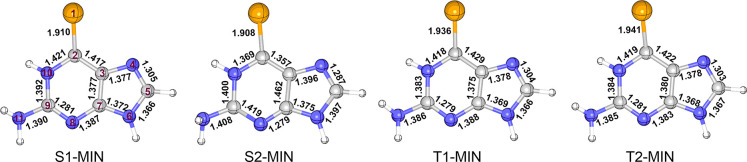
Minimum-energy structures of 6SeG in water solution optimized at the QM(CASSCF)/MM level of theory. Selected bond lengths are in Å.

Figure 4 shows the lowest-lying minimum-energy structures optimized in aqueous solution using the QM(CASSCF)/MM method. The S_1_ minimum (S1-MIN) is located 2.44 eV adiabatically above the S_0_ minimum at the QM1(MS-CASPT2)/MM level of theory. It is characterized by a pyramidalization of the Se atom (by 24°) and an elongated C=Se bond length (by 0.12 Å) with respect to the S_0_ minimum. The S2-MIN is predicted at 3.34 eV above the S_0_ minimum. It retains the Se atom in the molecular plane and also shows a stretched C=Se bond (by 0.118 Å) in comparison to the S_0_ minimum, while the amino group is rotated making its two hydrogen atoms almost perpendicular to the molecular plane.

The T1-MIN and T2-MIN also have the Se atom pyramidalized by about 30°. The C=Se bond length is longer in the T_2_ minimum than in the T_1_ (1.941 vs 1.936 Å). These minima are calculated 2.38 and 2.45 eV above the S_0_ minimum. Experimentally, the triplet state lifetime in 6SeGua is reported to be 835 times shorter than that observed for 6tG in aqueous solution.^34^ Earlier theoretical investigations in 6tG pointed out the existence of two minimum structures in the T_1_ state,^81^ but only a single minimum is predicted for 6SeGua. Hence, we suggest that the absence of the second minimum on T_1_ PES could be one of the reasons for the shorter triplet state population.

For completeness, these minima have also been optimized with the PCM model. In general, the results are very similar to those obtained with QM/MM but the pyramidalization on the Se atom becomes larger (S1-MIN, 35°; S2-MIN, 44°; T1-MIN, 42°; T2-MIN, 41°). The observed larger pyramidalization angles in relation to those computed with the QM/MM method can be explained by considering how the solute–solvent hydrogen bonds are modeled in each approach. In addition, the adiabatic excitation energies are also influenced, with the PCM model predicting values of 2.99, 3.18, 2.40, and 2.71 eV for the S1-MIN, S2-MIN, T1-MIN, and T2-MIN structures, respectively. Overall, in the particular case of 6SeG, both solvation models well agree about the relative energy order of the minima.

**Figure 5:**
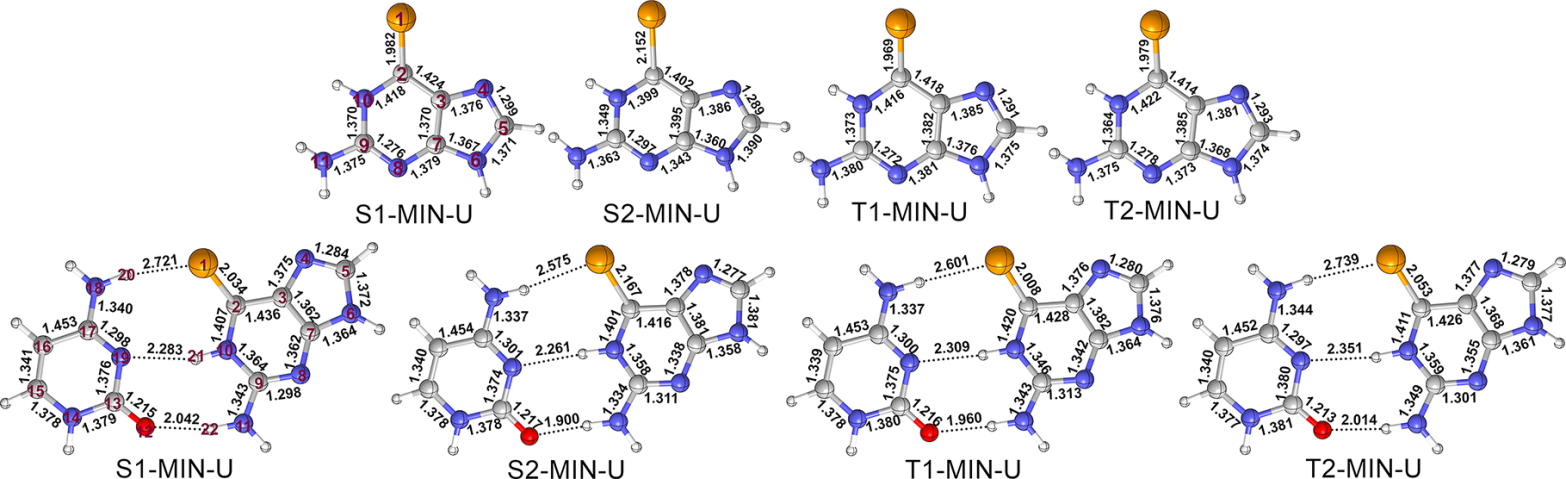
Minimum-energy structures of 6SeG in the S_2_, S_1_, T_2_, and T_1_ states in DNA optimized at QM1(CASSCF)/MM (top) and QM2(CASSCF)/MM (down) levels of theory. In these structures, the C=Se group is twisted out of the molecular plane, which is referred to as “up” configuration and indicated by “U” in the structure labels (see “central” and “down” in Figures S5–S8). Selected bond lengths are in Å.

The excited-state minimum-energy structures have also been optimized in DNA at the QM1 and QM2(CASSCF)/MM levels of theory; see Figure 5. The corresponding S_1_, S_2_, T_1_, and T_2_ minima can be classified into three groups, differing by the orientation of the C=Se group–above (“up”), below (“down”) or within the molecular plane (“central”), denoted as U, D, and C, respectively. The Se atom leaves the molecular plane pointing toward the guanine in the “up” structure, and pointing toward the thymine in the “down” structure (see Figure S9). As they are energetically quasi-degenerated (Tables S3 and S4), only the “up” conformations (referred as S1-MIN-U, S2-MIN-U, T1-MIN-U, and T2-MIN-U) will be discussed.

Conspicuously, the QM1(CASSCF)/MM calculations predict a C=Se bond length elongated in all the structures, by more than 0.30 Å in S2-MIN-U and by about 0.15 Å in the other minima, with respect to the S_0_ minimum. In comparison, the other bond lengths change less than 0.03 Å. The calculations with QM2(CASSCF)/MM are consistent, with changes in the C=Se bond ranging from 0.15 to 0.32 Å and the other bond differing by less than 0.03 Å. This implies that the presence of the pairing cytosine hardly influences the geometries of the 6SeG moiety. Interestingly, the electronic excitations obtained with both QM regions are also very similar, as it has been also observed in other nonnatural base pairs in DNA.^83^ The adiabatic excitation energies of the S_1_, S_2_, T_1_, and T_2_ are to be, 2.46 (2.58), 3.08 (2.86), 2.27 (2.29), and 2.36 (2.53) eV at the QM1(MS-CASPT2)/MM level (QM2(MS-CASPT2)/MM in parentheses). Structures and energies of the other conformations can be found in Figures S5–S8 and Tables S4 and S5.

### 3.4 Crossing Points

Crossing points among different PES are key to disentangling excited-state decay pathways. Unlike excited-state minima, the optimization of crossing points is much more difficult. Here, we have first optimized two-state crossing points at the QM(CASSCF)/MM level of theory and after analyzing the nature of the involved electronic states, a single-point energy calculation was done with QM(MS-CASPT2)/MM, verifying whether the two states keep degenerated. In some cases, some two-state minimum-energy crossing points resulted in three-state degenerate points.

**Figure 6:**
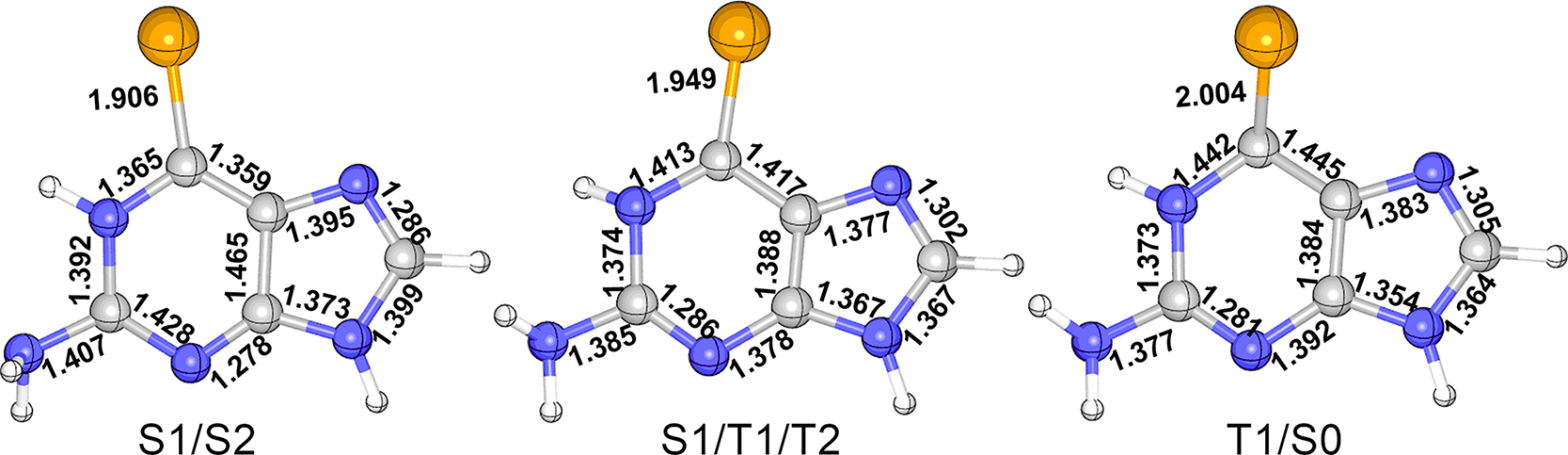
Minimum crossing points of 6SeG in solution obtained with QM1(CASSCF)/MM. Selected bond lengths are in Å.

In water, three minimum-energy crossing points have been found; see Figure 6. The first one corresponds to a crossing between the S_2_ and S_1_ states (S2/S1). This conical intersection is very similar to the S2-MIN; as a matter of fact, both structures are quasi-degenerate in energy (S2-MIN lies at 3.34 eV while S2/S1 is at 3.34/3.29 eV). For this reason, we computed numerical frequencies at the SA-CASSCF level of theory (the same level of theory employed for optimizations) in all environments, to make sure that it is a true minimum and not simply the lowest point on the S_1_/S_2_ intersection seam. Similar to the three-state crossing point found for 6SeG in gas phase,^47^ as well as for other canonical and modified nucleobases either in gas phase or in solution,^84−87^ the search for a conical intersection between the two low-lying T_1_ and T_2_ states, returned a three-state degenerate crossing point on which the S_1_ state is also degenerated. This S1/T1/T2 three-state crossing structure has the Se atom out of the molecular plane by ca. 28° and a C=Se bond length of 1.949 Å.

The last crossing point found in aqueous solution is the singlet/triplet crossing T1/S0, between the S_0_ and T_1_ PES. This structure shows a distinctive large pyramidalization at the Se atom (ca. 61°)—larger than in any of the other optimized critical points.

**Figure 7:**
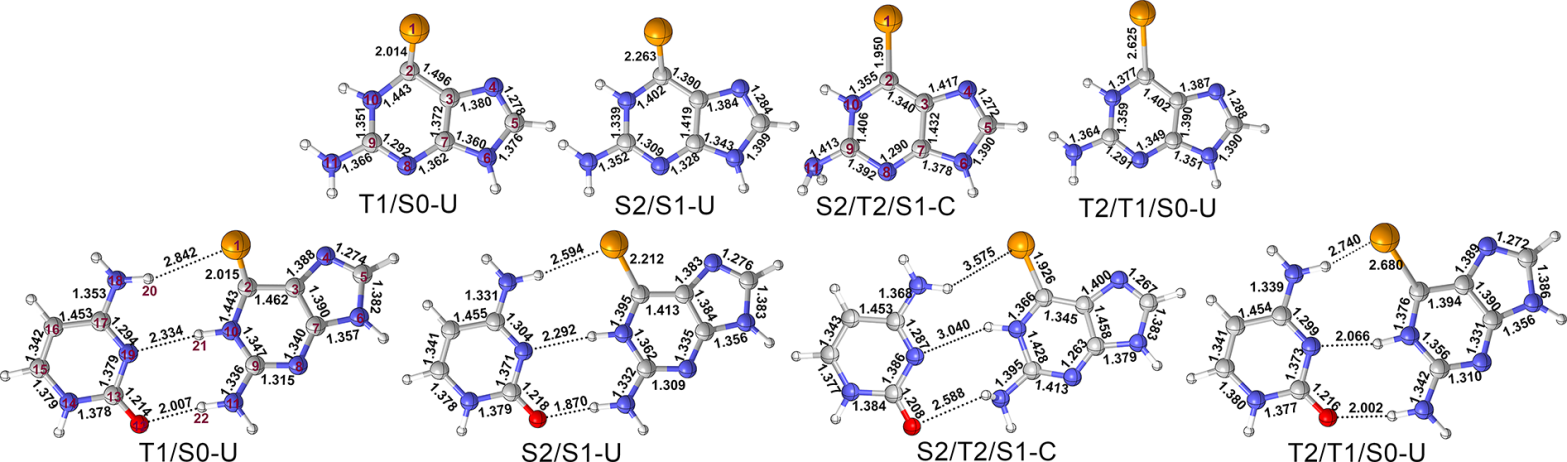
Minimum crossing points of 6SeG in DNA obtained with QM1(CASSCF)/MM (top) and QM2(CASSCF)/MM (down) levels of theory. Selected bond lengths are in Å.

Figure 7 shows the obtained crossing points of 6SeG in DNA; as with the local minima, only the “up” structures are shown, unless otherwise stated (see Figures S10 and S11 for other conformations). The two S_2_/S_1_ conical intersections (S2/S1-U) are very similar, except for C=Se bond and its orientation relative to the molecular plane. As expected, the presence of the hydrogen bond shortens the C=Se bond (from 2.263 to 2.212 Å); however, in comparison, changes in the other bond lengths can be disregarded. The energetics of the S2/S1-U geometries are also affected by the consideration of the base pair (see Table S5). Accordingly, while the S_2_ and S_1_ states are at 3.28 and 3.20 eV for QM1(MS-CASPT2)/MM, they are stabilized to 2.90 and 2.86 eV for QM2(MS-CASPT2)/MM; see Table S2.

Singlet/triplet T_1_/S_0_ crossing points were also optimized for 6SeG in DNA. The T1/S0-U shows a C=Se bond length much longer than that in the S_0_ minimum (2.004 (QM1)/2.015 (QM2) Å in the T1/S0-U versus 1.827 (QM1)/1.848 Å in S0-MIN). The S_0_ and T_1_ energies at T1S0-U were calculated to be 2.58 and 2.62 eV (QM1) and 2.43 and 2.40 eV (QM2), i.e., in contrast to the S2/S1-U conical intersection. Here, the presence of cytosine has a negligible effect on the energies.

In addition to the two-state intersection structures, two different three-state intersection structures were also identified for 6SeG in DNA: the S2/S1/T2-C and the T2/T1/S0-U. The S2/S1/T2-C crossing structure is characterized by a much longer C=Se bond length (1.950 and 1.926 Å) and a Se–N11–N4–N6 dihedral angle (180° and 175°), considering QM1 and QM2 regions, respectively. The most striking structural feature of S2/T2/S1-C, with respect to the other intersection structures, is that the NH_2_ group becomes nearly perpendicular to the molecular plane. Moreover, the C=Se bond is essentially in the molecular plane, unlike the more pyramidalized feature of S2/S1-U and T1/S0-U. The energies of the S2/S1/T2-C structure are 4.11/4.07/4.10 eV at the best QM2(MS-CASPT2)/MM level of theory, indicating that this crossing point is not accessible.

The T2/T1/S0 crossing points differ on the orientation of the C=Se, and accordingly, they are labeled as T2T1S0-U and T2T1S0-D. From them, only T2/T1/S0-U is shown in Figure 7 (see Figure S10 for the -D conformation). As can be seen, T2/T1/S0-U presents a longer C=Se bond length (2.625(QM1) or 2.680(QM2) Å) than in the other crossing points. The estimated energies of the T_2_/T_1_/S_0_ states are respectively 3.06/2.96/3.03 eV or 3.04, 3.03, and 2.97 eV with the smaller QM1 region, showing that the hydrogen bonding interaction has very small effects on the energetics.

### 3.5 Excited-State Relaxation Pathways

**Figure 8:**
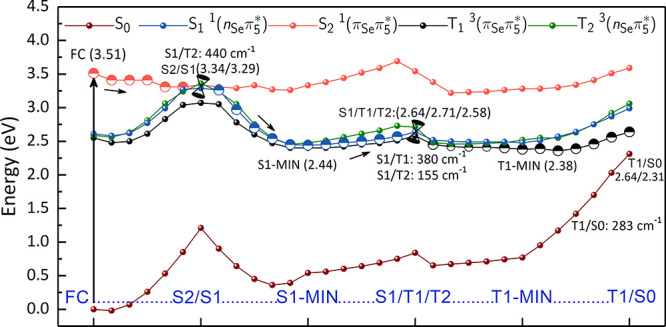
Plausible relaxation pathway of 6SeG in water calculated at the QM(MS-CASPT2)/MM level of theory. Relative energies of minima and crossing points are in eV. Spin–orbit couplings at the singlet/triplet crossings are given in cm^–1^.

Finally, having obtained all the important critical points of 6SeG in water and in DNA, we proceed now to discuss the excited-state relaxation mechanisms, assisted by the linear interpolation internal coordinates (LIIC) technique. Note that even when the energy barriers computed with the LIIC technique are typically overestimated, these scans allows us to postulate the most plausible deactivation mechanisms, which can be further confirmed only by means of a detailed nonadiabatic molecular dynamics study. Additionally, linear interpolation in internal coordinates scans allow us to verify the consistency of the active space employed for the computation of all points, whether or not there are energy barriers due to intermediate geometries connecting the initial and final points, and to enable a graphical presentation which resembles the familiar potential energy surface concept. Figure 8 displays the obtained pathways for 6SeG in water.

According to our results, the primary UV absorption event populates the bright S_2_ (^1^π_Se_π_5_^*^) state. After that, the system evolves barrierless along the S_2_ PES toward the S2/S1 conical intersection, by dissipating 0.17 eV excess of energy. Such an energetic profile is also confirmed using the minimum-energy path technique with QM(CASSCF)/MM (see Figure S12). The S2/S1 conical intersection coincides with the S2-MIN that simultaneously is also a three-state intersection, where the S_1_, S_2_, and T_2_ electronic states are quasi-degenerated. Therefore, in the vicinity of S2-MIN, the S_2_ electronic population can be transferred to both S_1_ and T_2_ states, through a conical intersection or a singlet/triplet crossing, respectively. The latter process benefits from a large spin–orbit coupling of ca. 440 cm^–1^ between S_2_ and T_2_. However, which of these pathways is preferred should be elucidated with the help of dynamical simulations.

Presuming a partial population transfer from the S_2_ to S_1_ state, the system can then reach the S_1_ minimum (S1-MIN), located 0.90 eV below the S2/S1 conical intersection region, as suggested by the downhill LIIC pathway. Starting from the S1-MIN region, we investigate whether the S1/T2/T1 region, located only 0.17 eV above the S1-MIN region, could be accessed. The computed LIIC profile does not show any barrier along this path and taking into account that the SOC is also substantial (S_1_/T_1_ = 380 cm^–1^ and S_1_/T_2_ = 155 cm^–1^), we hypothesize that population transfer to the T_1_ state should be favorable. In principle, internal conversion to the ground state and the ISC process could occur in parallel. However, the optimized minimum energy crossing point between the S_1_ and the ground state was computed to be located 1 eV above the S1 min region. Based on that, the direct relaxation pathway from the S_1_ to the S_0_ state is suppressed, in agreement with what is observed in other similar systems.^16,48^ Therefore, direct relaxation pathways to the S_0_ were not considered by us.

The effects of sulfur-to-selenium substitution in the photophysics promotes a substantial decrease of the triplet lifetime, as observed by the experiment.^34^ Our LIIC scans shows that the ISC process is favorable for 6SeG in water; however, from static calculations, we are not able to quantify how much more efficient this process is in relation to 6tG. Thus, only nonadiabatic dynamics simulations could be used in order to better understand why the 6SeG triplet lifetime is shorter. From here, there is a barrierless pathway toward the T1-MIN (0.26 eV below the three-state S1/T2/T1 crossing region), from which radiative and nonradiative processes can occur. The system can emit phosphorescence at 1.63 eV. Alternatively, as shown by the LIIC pathway, the singlet/triplet T1/S0 crossing located adiabatically 0.26 eV above the T_1_ minimum, allows an intersystem crossing to the S_0_ state. The calculated spin–orbit coupling for this crossing (283 cm^–1^), despite smaller than the previous values, could be sufficient for an efficient deactivation. Although, with MS-CASPT2 the energy gap at the T1/S0 crossing is still 0.33 eV at the CASSCF optimized structure, we assume that population to the ground state is viable. For comparison, in gas phase, the T1/S0 crossing point is predicted 0.11 eV above the T1-MIN at the MS-CASPT2 level of theory^47^ – a little lower than that in aqueous solution.

**Figure 9:**
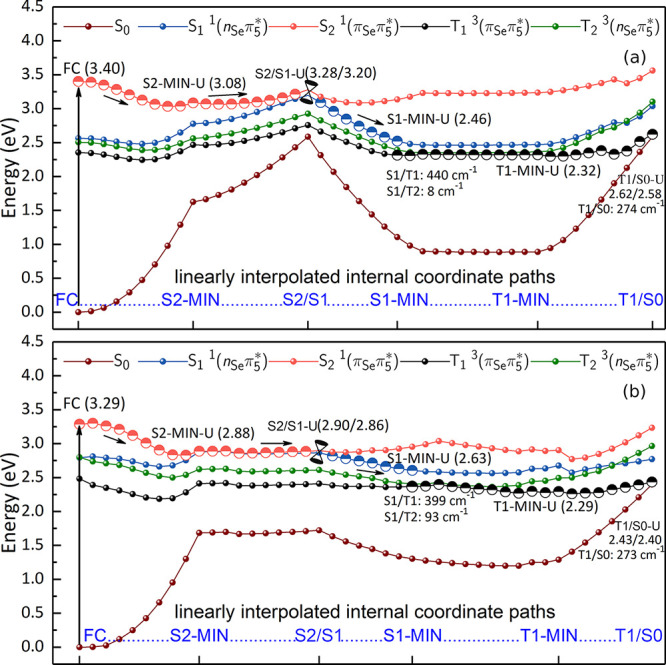
Plausible relaxation pathway of 6SeG in DNA calculated at the (a) QM1(MS-CASPT2)/MM and (b) QM2(MS-CASPT2)/MM levels of theory. Relative energies of minima and crossing points are in eV. Spin–orbit couplings at the singlet/triplet crossings are given in cm^–1^.

The excited-state deactivation mechanisms of 6SeG (QM1) and 6SeG-Cyt (QM2) in DNA have been also investigated, see Figure 9. In DNA, three possible deactivation mechanisms can be followed from the Franck–Condon region. One of them involves a barrierless relaxation to the S_2_ minimum with the *up* configuration i.e. S2-MIN-U, by releasing 0.32 eV of energy, as computed at the QM1(MS-CASPT2)/MM level (Figure 9a). Once in the S2-MIN-U region, the system evolves toward an internal conversion region with the S_1_ state (S2/S1-U, with the *up* configuration too, which is located adiabatically 0.20 eV above the S2-MIN-U region. From here, population is transferred to the S_1_ state, finally reaching barrierlessly the S_1_ minimum S1-MIN-U, in which the *up* configuration is kept. Analogously, compared to the 6SeG in water, the S_1_ of 6SeG and 6SeG-Cyt in DNA need to overcome 1.03 and 0.67 eV energy barriers to reach the S1/S0 conical intersection region, whereas LIIC paths connecting the S_1_ and triplet manifolds are nearly barrierless; consequently, we believe that S_1_ directly deactivating to the ground state is inefficient. The S_1_ minimum region also represents a three-state crossing region among the S_1_, T_2_, and T_1_ electronic states, on which the population can be transferred from the S_1_ to T_1_ or T_2_ states, via intersystem crossings (S1/T1 or S1/T2). The S_1_ → T_1_ intersystem crossing process is more likely due to its larger S_1_/T_1_ spin–orbit coupling of 439.4 cm^–1^, in accord to the El-Sayed rule.^91^ Once on the T_1_ state, the system evolves to T1-MIN-U, from which the intersystem crossing with the S_0_ state, T_1_/S_0_ (T1/S0-U, SOC = 273 cm^–1^), can be reached after overcoming an energetic barrier of ca. 0.30 eV. Due to this barrier, the system will be trapped a while in the T_1_ state, before hopping to the S_0_ state.

Alternatively, the S_2_ state can also relax toward another S_2_ minimum, the S2-MIN-D (see Figure S13). In such a case, and as discussed previously, after reaching the T1-MIN-D, it is necessary to overcome an energy barrier of 0.26 eV to access the T1/S0-D crossing point. Finally, if instead, the S_2_ state relaxes to the S2-MIN-C minimum, its further relaxation to the S_1_ state via the S2/S1/T2-C three-state intersection point encounters a comparable barrier in the S_2_ state because this process involves a large rotation of the N11H_2_ group (see Figure S14). Accordingly, this pathway does not compete with the other two. However, if S2-MIN-C can be first converted either into S2-MIN-U or S2-MIN-D in the S_2_ state, the system initially populated at S2-MIN-C still can decay to the T_1_ state efficiently.

The calculations of the excited-state relaxation pathways of 6SeG in DNA using the GC base pair (Figure 9b) are similar to those described above, with few exceptions. The S_2_ potential energy is much steeper, so that the initial S_2_ relaxation pathway toward its minimum, S2-MIN-U, should be faster. The internal conversion process from the S_2_ to S_1_ states via S2/S1-U is essentially barrierless (0.02 eV here versus 0.20 eV without C). This is mainly attributed to the hydrogen bond interaction between the 6SeG and C. Intermolecular hydrogen bonds stabilize the 6SeG ππ* excited state level, which significantly reduces the excited state lifetime. In fact, several similar phenomena were reported in the literature.^88−90^ For example, Temps et al. observed that the formation of guanine and cytosine Watson–Crick base pairs results in ultrafast nonradiative transition.^88^ Around the S1-MIN-U minimum, the system will undergo intersystem crossing to both T_2_ and T_1_ states. But, as mentioned above, the S_1_ → T_1_ intersystem crossing is preferred because it has a larger S_1_/T_1_ spin–orbit coupling of 399 cm^–1^, justified by the El-Sayed rule.^91^ Once in the T_1_ state, the barrier separating T1-MIN-U from T1/S0-U increases to 0.14 eV, i.e., 0.14 eV higher than that without C. For completeness, we mention that when the S_2_ system relaxes to its other minimum S2-MIN-D, similar excited-state relaxation pathways are identified (see Figure S15) and the corresponding pathways from S2-MIN-C are not studied here because of the high energies of the S2/T2/S1–C triplet crossing point, which makes it unlikely (see above).

**Figure 10:**
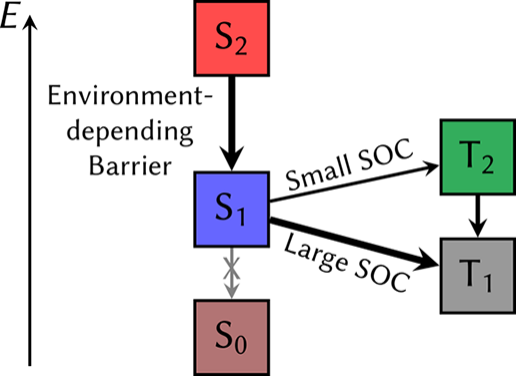
Possible deactivation mechanisms of 6SeG in gas phase, water, and DNA.

Figure 10 displays a schematic summary of our proposed relaxation mechanisms. In short, the environment surrounding the 6SeG greatly affects how fast the internal conversion from the bright state to the lowest singlet state takes place. A high energy barrier is computed in the gas phase,^47^ disappearing in water. Depending on which structure will be primarily accessed in DNA, the population transfer to the S_1_ PES may be faster or slower. Afterward, the population will be easily transferred to the triplet states via ISC, since the internal conversion to the S_0_ is less important.

## 4 Conclusions

A multiscale QM/MM strategy including the very accurate MS-CASPT2 method for the QM part has been employed to explore the excited-state properties and excited-state decay pathways of 6SeG in water and in a DNA environment, and compare these to those in gas phase. In the DNA environment, the effect of the pairing cytosine was also investigated by including it into the QM region. Spectroscopically, in all the three environments, 6SeG is characterized by the same two lowest excited singlet states, i.e. S_1_^1^(n_Se_π_5_^*^) and S_2_^1^(π_Se_π_5_^*^), corresponding to a dark and the spectroscopically bright excited states, respectively. Two triplet electronic states, i.e., T_1_^3^(π_Se_π_5_^*^) and T_2_^3^(n_Se_π_5_^*^), were found to be energetically lower than the bright S_2_ state. We find that the order of the singlet and triplet states and their characters does not depend on the environment; moreover, the vertical excitation energies in water and DNA are very similar to each other, as long as only the 6SeG is considered quantum mechanically. The inclusion of explicit hydrogen-bonding interactions stabilizes the S_2_^1^(π_Se_π_5_^*^) state and destabilizes the S_1_^1^(n_Se_π_5_^*^) state.

For comparison, the inclusion of solvent is also taken into account by implicit methods to find that PCM model overestimates both S_1_^1^(n_Se_π_5_^*^) and T_2_^3^(n_Se_π_5_^*^) states, in relation to the values computed with the QM/MM method.

In water, we have found that the excited-state relaxation pathway corresponds to an efficient relaxation from the initially populated S_2_^1^(π_Se_π_5_^*^) state to the T_1_^3^(π_Se_π_5_^*^) state. After irradiation, the S_2_ state quickly relaxes from the Franck–Condon region to an S_2_ minimum, near which a S_2_/S_1_/T_2_ three-state intersection region induces both S_2_ →S_1_ internal conversion and S_2_ → T_2_ intersystem crossing processes. Subsequently, it is expected that both the S_1_ or T_2_ states decay further to the T_1_ state via either S_1_ → T_1_ intersystem crossing or T_2_ → T_1_ internal conversion. Both intersystem crossing processes benefit from substantial spin–orbit couplings (S_1_/T_2_ = 440 cm^–1^ and S_1_/T_1_ = 380 cm^–1^). Once in the T_1_ state, the system should be trapped for some time, due to the existence of a small energy barrier between the T_1_ minimum and the T_1_/S_0_ crossing point, estimated to be 0.26 eV.

The inclusion of DNA environment increases the complexity of the excited-state relaxation pathways of 6SeG due to the existence of different conformational structures, that depend on whether the C=Se group is oriented above (“up”), below (“down”) or within (“central”) the molecular plane. In general, it is possible to identify a preferred pathway, with resemblances to that in water. For example, starting from the up configuration, the excited S_2_ system first arrives at its S_2_ minimum, and then, a nearby S_2_/S_1_ conical intersection drives the system down to the S_1_ state. An S_1_ → T_1_ intersystem crossing process then occurs in the vicinity of the S_1_ minimum, and an energy barrier of 0.30 eV traps the system in the T_1_ state before it can go to the S_0_ state. A similar excited-state decay pathway is found from the down configuration, while the central configuration is predicted not to be efficient because of a large energy barrier in the S_2_ state. Which of these pathways is most probable and in which amounts could be predicted by dynamical simulations.

The inclusion of the pairing cytosine has little influence on the obtained relaxation mechanism, except that the S_2_ → S_1_ internal conversion processes is predicted to be faster and larger energy gaps between the T_1_ minima and the T_1_/S_0_ crossing points are found.

The present work provides an important step toward understanding the excited state properties of chemically modified nucleobases and its response to light, on the way to find better photosensitizers, for example for phototherapies.

## Supporting Information Available

Minima and intersection structures for the other structures optimized in DNA, excited state relaxation paths for the down (D) and central (C) structures optimized in DNA, simulated electronic absorption spectrum in water, minimum energy path in water, and Cartesian coordinates of the optimized structures (PDF)

## References

[ref1] Schreier W. J.; Gilch P.; Zinth W. Early Events of DNA Photodamage. Annu. Rev. Phys. Chem. 2015, 66, 497–519. 10.1146/annurev-physchem-040214-121821. 25664840

[ref2] Kang H.; Lee K. T.; Jung B.; Ko Y. J.; Kim S. K. Intrinsic Lifetimes of the Excited State of DNA and RNA Bases. J. Am. Chem. Soc. 2002, 124, 12958–12959. 10.1021/ja027627x. 12405817

[ref3] Cohen B.; Hare P. M.; Kohler B. Ultrafast Excited-State Dynamics of Adenine and Monomethylated Adenines in Solution: Implications for the Nonradiative Decay Mechanism. J. Am. Chem. Soc. 2003, 125, 13594–13601. 10.1021/ja035628z. 14583057

[ref4] Crespo-Hernández C. E.; Cohen B.; Hare P. M.; Kohler B. Ultrafast Excited-State Dynamics in Nucleic Acids. Chem. Rev. 2004, 104, 1977–2019. 10.1021/cr0206770. 15080719

[ref5] Tuna D.; Sobolewski A. L.; Domcke W. Mechanisms of Ultrafast Excited-State Deactivation in Adenosine. J. Phys. Chem. A 2014, 118, 122–127. 10.1021/jp410121h. 24320624

[ref6] Blancafort L. Excited-State Potential Energy Surface for the Photophysics of Adenine. J. Am. Chem. Soc. 2006, 128, 210–219. 10.1021/ja054998f. 16390149

[ref7] Middleton C. T.; de La Harpe K.; Su C.; Law Y. K.; Crespo-Hernández C. E.; Kohler B. DNA Excited-State Dynamics: From Single Bases to the Double Helix. Annu. Rev. Phys. Chem. 2009, 60, 217–239. 10.1146/annurev.physchem.59.032607.093719. 19012538

[ref8] Barbatti M.; Aquino A. J. A.; Szymczak J. J.; Nachtigallová D.; Hobza P.; Lischka H. Relaxation Mechanisms of UV-Photoexcited DNA and RNA Nucleobases. Proc. Natl. Acad. Sci. U. S. A. 2010, 107, 21453–21458. 10.1073/pnas.1014982107. 21115845 PMC3003128

[ref9] Giussani A.; Segarra-Martí J.; Roca-Sanjuán D.; Merchán M. In Photoinduced Phenomena in Nucleic Acids I: Nucleobases in the Gas Phase and in Solvents; Barbatti M., Borin A. C., Ullrich S., Eds.; Springer International Publishing: Cham, Switzerland, 2015; pp 57–97.

[ref10] Mai S.; Richter M.; Marquetand P.; González L. In Photoinduced Phenomena in Nucleic Acids I: Nucleobases in the Gas Phase and in Solvents; Barbatti M., Borin A. C., Ullrich S., Eds.; Springer International Publishing: Cham, Switzerland, 2015; pp 99–153.

[ref11] Improta R.; Santoro F.; Blancafort L. Quantum Mechanical Studies on the Photophysics and the Photochemistry of Nucleic Acids and Nucleobases. Chem. Rev. 2016, 116, 3540–3593. 10.1021/acs.chemrev.5b00444. 26928320

[ref12] Matsika S. In Photoinduced Phenomena in Nucleic Acids I: Nucleobases in the Gas Phase and in Solvents; Barbatti M., Borin A. C., Ullrich S., Eds.; Springer International Publishing: Cham, Switzerland, 2015; pp 209–243.

[ref13] Harada Y.; Suzuki T.; Ichimura T.; Xu Y.-Z. Triplet Formation of 4-Thiothymidine and Its Photosensitization to Oxygen Studied by Time-Resolved Thermal Lensing Technique. J. Phys. Chem. B 2007, 111, 5518–5524. 10.1021/jp0678094. 17439266

[ref14] Kuramochi H.; Kobayashi T.; Suzuki T.; Ichimura T. Excited-State Dynamics of 6-Aza-2-Thiothymine and 2-Thiothymine: Highly Efficient Intersystem Crossing and Singlet Oxygen Photosensitization. J. Phys. Chem. B 2010, 114, 8782–8789. 10.1021/jp102067t. 20552955

[ref15] Harada Y.; Okabe C.; Kobayashi T.; Suzuki T.; Ichimura T.; Nishi N.; Xu Y.-Z. Ultrafast Intersystem Crossing of 4-Thiothymidine in Aqueous Solution. J. Phys. Chem. Lett. 2010, 1, 480–484. 10.1021/jz900276x.

[ref16] Mai S.; Pollum M.; Martínez-Fernández L.; Dunn N.; Marquetand P.; Corral I.; Crespo-Hernández C. E.; González L. The Origin of Efficient Triplet State Population in Sulfur-Substituted Nucleobases. Nat. Commun. 2016, 7, 13077 10.1038/ncomms13077. 27703148 PMC5059480

[ref17] Ashwood B.; Pollum M.; Crespo-Hernández C. E. Photochemical and Photodynamical Properties of Sulfur-Substituted Nucleic Acid Bases. Photochem. Photobiol. 2019, 95, 33–58. 10.1111/php.12975. 29978490

[ref18] Reichardt C.; Guo C.; Crespo-Hernández C. E. Excited-State Dynamics in 6-Thioguanosine from the Femtosecond to Microsecond Time Scale. J. Phys. Chem. B 2011, 115, 3263–3270. 10.1021/jp112018u. 21384813

[ref19] Pollum M.; Crespo-Hernández C. E. Communication: The Dark Singlet State as a Doorway State in the Ultrafast and Efficient Intersystem Crossing Dynamics in 2-Thiothymine and 2-Thiouracil. J. Chem. Phys. 2014, 140, 071101 10.1063/1.4866447. 24559331

[ref20] Mohamadzade A.; Bai S. M.; Barbatti M.; Ullrich S. Intersystem Crossing Dynamics in Singly Substituted Thiouracil Studied by Time-Resolved Photoelectron Spectroscopy: Micro-Environmental Effects due to Sulfur Position. Chem. Phys. 2018, 515, 572–579. 10.1016/j.chemphys.2018.08.011.

[ref21] Ashwood B.; Jockusch S.; Crespo-Hernández C. E. Excited-State Dynamics of the Thiopurine Prodrug 6-Thioguanine: Can N9-Glycosylation Affect Its Phototoxic Activity?. Molecules 2017, 22, 379 10.3390/molecules22030379. PMC6155220 28264514

[ref22] Oniszczuk A.; Wojtunik-Kulesza K. A.; Oniszczuk T.; Kasprzak K. The potential of photodynamic therapy (PDT) - Experimental investigations and clinical use. Biomed. Pharmacother. 2016, 83, 912–929. 10.1016/j.biopha.2016.07.058. 27522005

[ref23] Pollum M.; Jockusch S.; Crespo-Hernández C. E. 2,4-Dithiothymine as a Potent UVA Chemotherapeutic Agent. J. Am. Chem. Soc. 2014, 136, 17930–17933. 10.1021/ja510611j. 25506742

[ref24] Pollum M.; Ortiz-Rodríguez L. A.; Jockusch S.; Crespo-Hernández C. E. The Triplet State of 6-thio-2’-deoxyguanosine: Intrinsic Properties and Reactivity Toward Molecular Oxygen. Photochem. Photobiol. 2016, 92, 286–292. 10.1111/php.12563. 26757207

[ref25] Pollum M.; Lam M.; Jockusch S.; Crespo-Hernández C. E. Dithionated nucleobases as effective photodynamic agents against human epidermoid carcinoma cells. ChemMedChem 2018, 13, 1044–1050. 10.1002/cmdc.201800148. 29532623

[ref26] Mautner H. G.; Chu S.-H.; Jaffe J. J.; Sartorelli A. C. The Synthesis and Antineoplastic Properties of Selenoguanine, Selenocytosine and Related Compounds. J. Med. Chem. 1963, 6, 36–39. 10.1021/jm00337a008. 14174027

[ref27] Aboul-Enein H. Y.; Awad A. A.; Al-Andis N. M. Synthesis and the antiperoxidase activity of seleno analogues of the antithyroid drug propylthiouracil. J. Enzyme Inhib. 1993, 7, 147–150. 10.3109/14756369309040756. 7509869

[ref28] Caton-Williams J.; Huang Z. Biochemistry of Selenium-Derivatized Naturally Occurring and Unnatural Nucleic Acids. Chem. Biodiversity 2008, 5, 396–407. 10.1002/cbdv.200890040. 18357549

[ref29] Caton-Williams J.; Huang Z. Synthesis and DNA-Polymerase Incorporation of Colored 4-Selenothymidine Triphosphate for Polymerase Recognition and DNA Visualization. Angew. Chem. 2008, 120, 1747–1749. 10.1002/ange.200705213. 18203229

[ref30] Salon J.; Gan J.; Abdur R.; Liu H.; Huang Z. Synthesis of 6-Se-guanosine RNAs for structural study. Org. Lett. 2013, 15, 3934–3937. 10.1021/ol401698n. 23859218 PMC3763942

[ref31] Hassan A. E.; Sheng J.; Zhang W.; Huang Z. High fidelity of base pairing by 2-selenothymidine in DNA. J. Am. Chem. Soc. 2010, 132, 2120–2121. 10.1021/ja909330m. 20108896

[ref32] Faustino I.; Curutchet C.; Luque F. J.; Orozco M. The DNA-forming properties of 6-selenoguanine. Phys. Chem. Chem. Phys. 2014, 16, 1101–1110. 10.1039/C3CP53885K. 24287926

[ref33] Manae M. A.; Hazra A. Interplay between conjugation and size-driven delocalization leads to characteristic properties of substituted thymines. J. Phys. Chem. A 2017, 121, 8147–8153. 10.1021/acs.jpca.7b08566. 28960980

[ref34] Farrell K. M.; Brister M. M.; Pittelkow M.; Sølling T. I.; Crespo-Hernández C. E. Heavy-Atom-Substituted Nucleobases in Photodynamic Applications: Substitution of Sulfur with Selenium in 6-Thioguanine Induces a Remarkable Increase in the Rate of Triplet Decay in 6-Selenoguanine. J. Am. Chem. Soc. 2018, 140, 11214–11218. 10.1021/jacs.8b07665. 30145892

[ref35] Mai S.; González L. Molecular Photochemistry: Recent Developments in Theory. Angew. Chem., Int. Ed. 2020, 59, 16832–16846. 10.1002/anie.201916381. PMC7540682 32052547

[ref36] Bai S.; Barbatti M. On the decay of the triplet state of thionucleobases. Phys. Chem. Chem. Phys. 2017, 19, 12674–12682. 10.1039/C7CP02050C. 28474025

[ref37] Cui G. L.; Thiel W. Intersystem Crossing Enables 4-Thiothymidine to Act as a Photosensitizer in Photodynamic Therapy: An Ab Initio QM/MM Study. J. Phys. Chem. Lett. 2014, 5, 2682–2687. 10.1021/jz501159j. 26277963

[ref38] Cui G. L.; Fang W. H. State-Specific Heavy-Atom Effect on Intersystem Crossing Processes in 2-Thiothymine: A Potential Photodynamic Therapy Photosensitizer. J. Chem. Phys. 2013, 138, 044315 10.1063/1.4776261. 23387592

[ref39] Gobbo J. P.; Borin A. C. 2-Thiouracil Deactivation Pathways and Triplet States Population. Comput. Theor. Chem. 2014, 1040-1041, 195–201. 10.1016/j.comptc.2014.03.021.

[ref40] Gobbo J. P.; Borin A. C. On the Population of Triplet Excited States of 6-Aza-2-Thiothymine. J. Phys. Chem. A 2013, 117, 5589–5596. 10.1021/jp403508v. 23777466

[ref41] Yu H.; Sanchez-Rodriguez J. A.; Pollum M.; Crespo-Hernández C. E.; Mai S.; Marquetand P.; González L.; Ullrich S. Internal Conversion and Intersystem Crossing Pathways in UV Excited, Isolated Uracils and Their Implications in Prebiotic Chemistry. Phys. Chem. Chem. Phys. 2016, 18, 20168–20176. 10.1039/C6CP01790H. 27189184

[ref42] Mai S.; Marquetand P.; González L. Intersystem Crossing Pathways in the Noncanonical Nucleobase 2-Thiouracil: A Time-Dependent Picture. J. Phys. Chem. Lett. 2016, 7, 1978–1983. 10.1021/acs.jpclett.6b00616. 27167106 PMC4893732

[ref43] Mai S.; Marquetand P.; González L. A Static Picture of the Relaxation and Intersystem Crossing Mechanisms of Photoexcited 2-Thiouracil. J. Phys. Chem. A 2015, 119, 9524–9533. 10.1021/acs.jpca.5b06639. 26284285 PMC4568544

[ref44] Ibele L. M.; Sánchez-Murcia P. A.; Mai S.; Nogueira J. J.; González L. Excimer Intermediates en Route to Long-Lived Charge-Transfer States in Single-Stranded Adenine DNA as Revealed by Nonadiabatic Dynamics. J. Phys. Chem. Lett. 2020, 11, 7483–7488. 10.1021/acs.jpclett.0c02193. 32794719 PMC7503858

[ref45] Warshel A.; Levitt M. Theoretical Studies of Enzymic Reactions - Dielectric, Electrostatic and Steric Stabilization of Carbonium-Ion in Reaction of Lysozyme. J. Mol. Biol. 1976, 103, 227–249. 10.1016/0022-2836(76)90311-9. 985660

[ref46] Senn H. M.; Thiel W. QM/MM Methods for Biomolecular Systems. Angew. Chem., Int. Ed. 2009, 48, 1198–1229. 10.1002/anie.200802019. 19173328

[ref47] Fang Y. G.; Peng Q.; Fang Q.; Fang W. H.; Cui G. L. MS-CASPT2 Studies on the Photophysics of Selenium-Substituted Guanine Nucleobase. ACS Omega 2019, 4, 9769–9777. 10.1021/acsomega.9b01276. 31460068 PMC6649137

[ref48] Mai S.; Wolf A. P.; González L. Curious Case of 2-Selenouracil: Efficient Population of Triplet States and Yet Photostable. J. Chem. Theory Comput. 2019, 15, 3730–3742. 10.1021/acs.jctc.9b00208. 31038951

[ref49] Peng Q.; Zhu Y. H.; Zhang T. S.; Liu X. Y.; Fang W. H.; Cui G. L. Selenium substitution effects on excited-state properties and photophysics of uracil: a MS-CASPT2 study. Phys. Chem. Chem. Phys. 2020, 22, 12120–12128. 10.1039/D0CP01369B. 32440669

[ref50] Case D.; Betz R.; Cerutti D. III; Darden T. C.; Duke T.; Giese R.; Gohlke T.; Goetz H.; Homeyer A.; Izadi N.; Amber 2016; University of California: San Francisco, CA, 2016.

[ref51] Pérez A.; Marchán I.; Svozil D.; Sponer J.; Cheatham T. E.; Laughton C. A.; Orozco M. Refinement of the AMBER Force Field for Nucleic Acids: Improving the Description of α/γ Conformers. Biophys. J. 2007, 92, 3817–3829. 10.1529/biophysj.106.097782. 17351000 PMC1868997

[ref52] Christofferson A.; Zhao L. F.; Sun H. Z.; Huang Z.; Huang N. Theoretical Studies of the Base Pair Fidelity of Selenium-Modified DNA. J. Phys. Chem. B 2011, 115, 10041–10048. 10.1021/jp204204x. 21770426

[ref53] Bayly C. I.; Cieplak P.; Cornell W.; Kollman P. A. A well-behaved electrostatic potential based method using charge restraints for deriving atomic charges: the RESP model. J. Phys. Chem. 1993, 97, 10269–10280. 10.1021/j100142a004.

[ref54] Vosko S. H.; Wilk L.; Nusair M. Accurate Spin-Dependent Electron Liquid Correlation Energies for Local Spin Density Calculations: A Critical Analysis. Can. J. Phys. 1980, 58, 1200–1211. 10.1139/p80-159.

[ref55] Becke A. D. Density-Functional Exchange-Energy Approximation with Correct Asymptotic Behavior. Phys. Rev. A: At., Mol., Opt. Phys. 1988, 38, 3098–3100. 10.1103/PhysRevA.38.3098. 9900728

[ref56] Lee C.; Yang W. T.; Parr R. G. Development of the Colle-Salvetti Correlation-Energy Formula into a Functional of the Electron Density. Phys. Rev. B: Condens. Matter Mater. Phys. 1988, 37, 785–789. 10.1103/PhysRevB.37.785. 9944570

[ref57] Becke A. D. A New Mixing of Hartree-Fock and Local Density-Functional Theories. J. Chem. Phys. 1993, 98, 1372–1377. 10.1063/1.464304.

[ref58] Dunning T. H. Jr. Gaussian Basis Sets for Use in Correlated Molecular Calculations. I. The Atoms Boron through Neon and Hydrogen. J. Chem. Phys. 1989, 90, 1007–1023. 10.1063/1.456153.

[ref59] Jorgensen W. L.; Chandrasekhar J.; Madura J. D.; Impey R. W.; Klein M. L. Comparison of Simple Potential Functions for Simulating Liquid Water. J. Chem. Phys. 1983, 79, 926–935. 10.1063/1.445869.

[ref60] Salon J.; Jiang J.; Sheng J.; Gerlits O. O.; Huang Z. Derivatization of DNAs with Selenium at 6-position of Guanine for Function and Crystal Structure Studies. Nucleic Acids Res. 2008, 36, 7009–7018. 10.1093/nar/gkn843. 18986998 PMC2602767

[ref61] Cheatham T. E.; Cieplak P.; Kollman P. A. A Modified Version of the Cornell et al. Force Field with Improved Sugar Pucker Phases and Helical Repeat. J. Biomol. Struct. Dyn. 1999, 16, 845–862. 10.1080/07391102.1999.10508297. 10217454

[ref62] Karlström G.; Lindh R.; Malmqvist P.-Å.; Roos B. O.; Ryde U.; Veryazov V.; Widmark P. O.; Cossi M.; Schimmelpfennig B.; Neogrady P.; Seijo L. MOLCAS: A Program Package for Computational Chemistry. Comput. Mater. Sci. 2003, 28, 222–229. 10.1016/S0927-0256(03)00109-5.

[ref63] Aquilante F.; Autschbach J.; Carlson R. K.; Chibotaru L. F.; Delcey M. G.; De Vico L.; Fdez. Galván I. F.; Ferré N.; Frutos L. M.; Gagliardi L.; et al. Molcas 8: New Capabilities for Multiconfigurational Quantum Chemical Calculations Across the Periodic Table. J. Comput. Chem. 2016, 37, 506–541. 10.1002/jcc.24221. 26561362

[ref64] Fdez. Galván I. F.; Vacher M.; Alavi A.; Angeli C.; Aquilante F.; Autschbach J.; Bao J. J.; Bokarev S. I.; Bogdanov N. A.; Carlson R. K.; et al. OpenMolcas: From Source Code to Insight. J. Chem. Theory Comput. 2019, 15, 5925–5964. 10.1021/acs.jctc.9b00532. 31509407

[ref65] Aquilante F.; Autschbach J.; Baiardi A.; Battaglia S.; Borin V. A.; Chibotaru L. F.; Conti I.; De Vico L.; Delcey M.; Fdez. Galván I.; et al. Modern quantum chemistry with [Open]Molcas. J. Chem. Phys. 2020, 152, 214117 10.1063/5.0004835. 32505150

[ref66] Rackers J. A.; Wang Z.; Lu C.; Laury M. L.; Lagardére L.; Schnieders M. J.; Piquemal J.-P.; Ren P.; Ponder J. W. Tinker 8: Software Tools for Molecular Design. J. Chem. Theory Comput. 2018, 14, 5273–5289. 10.1021/acs.jctc.8b00529. 30176213 PMC6335969

[ref67] Roos B. O. In Advances in Chemical Physics; Ab Initio Methods in Quantum Chemistry - II; Lawley K. P., Ed.; John Wiley & Sons Ltd.: Chichester, England, 1987; Vol. 69; pp 399–445.

[ref68] Andersson K.; Malmqvist P.-Å.; Roos B. O.; Sadlej A. J.; Wolinski K. Second-Order Perturbation Theory with a CASSCF Reference Function. J. Phys. Chem. 1990, 94, 5483–5488. 10.1021/j100377a012.

[ref69] Andersson K.; Malmqvist P.-Å.; Roos B. O. Second-Order Perturbation Theory with a Complete Active Space Self-Consistent Field Reference Function. J. Chem. Phys. 1992, 96, 1218–1226. 10.1063/1.462209.

[ref70] Wilson A. K.; Woon D. E.; Peterson K. A.; Dunning T. H. Gaussian Basis Sets for Use in Correlated Molecular Calculations. IX. The Atoms Gallium Through Krypton. J. Chem. Phys. 1999, 110, 7667–7676. 10.1063/1.478678.

[ref71] Försberg N.; Malmqvist P.-Å. Multiconfiguration Perturbation Theory with Imaginary Level Shift. Chem. Phys. Lett. 1997, 274, 196–204. 10.1016/S0009-2614(97)00669-6.

[ref72] Ghigo G.; Roos B. O.; Malmqvist P.-Å. A Modified Definition of the Zeroth-Order Hamiltonian in Multiconfigurational Perturbation Theory (CASPT2). Chem. Phys. Lett. 2004, 396, 142–149. 10.1016/j.cplett.2004.08.032.

[ref73] Zobel J. P.; Nogueira J. J.; González L. The IPEA dilemma in CASPT2. Chem. Sci. 2017, 8, 1482–1499. 10.1039/C6SC03759C. 28572908 PMC5452265

[ref74] Aquilante F.; Lindh R.; Bondo Pedersen T. B. Unbiased Auxiliary Basis Sets for Accurate Two-Electron Integral Approximations. J. Chem. Phys. 2007, 127, 114107–114713. 10.1063/1.2777146. 17887828

[ref75] Heß B. A.; Marian C. M.; Wahlgren U.; Gropen O. A Mean-Field Spin-Orbit Method Applicable to Correlated Wavefunctions. Chem. Phys. Lett. 1996, 251, 365–371. 10.1016/0009-2614(96)00119-4.

[ref76] Marian C. M.; Wahlgren U. A New Mean-Field and ECP-Based Spin-Orbit Method. Applications to Pt and PtH. Chem. Phys. Lett. 1996, 251, 357–364. 10.1016/0009-2614(95)01386-5.

[ref77] Marian C. M. Spin-Orbit Coupling and Intersystem Crossing in Molecules. Wiley Interdiscip. Rev. Comput. Mol. Sci. 2012, 2, 187–203. 10.1002/wcms.83.

[ref78] Barone V.; Cossi M. Quantum Calculation of Molecular Energies and Energy Gradients in Solution by a Conductor Solvent Model. J. Phys. Chem. A 1998, 102, 1995–2001. 10.1021/jp9716997.

[ref79] Tomasi J.; Mennucci B.; Cammi R. Quantum Mechanical Continuum Solvation Models. Chem. Rev. 2005, 105, 2999–3093. 10.1021/cr9904009. 16092826

[ref80] Xie B.-B.; Wang Q.; Guo W.-W.; Cui G. L. The Excited-State Decay Mechanism of 2,4-Dithiothymine in the Gas Phase, Microsolvated Surroundings, and Aqueous Solution. Phys. Chem. Chem. Phys. 2017, 19, 7689–7698. 10.1039/C7CP00478H. 28256672

[ref81] Martínez-Fernández L.; González L.; Corral I. An ab initio Mechanism for Efficient Population of Triplet States in Cytotoxic Sulfur Substituted DNA Bases: The Case of 6-thioguanine. Chem. Commun. 2012, 48, 2134–2136. 10.1039/c2cc15775f. 22245861

[ref82] Kistler K. A.; Matsika S. Photophysical pathways of cytosine in aqueous solution. Phys. Chem. Chem. Phys. 2010, 12, 5024–5031. 10.1039/b926125g. 20445904

[ref83] Wang Q.; Xie X.-Y.; Han J.; Cui G. L. QM and QM/MM Studies on Excited-State Relaxation Mechanisms of Unnatural Bases in Vacuo and Base Pairs in DNA. J. Phys. Chem. B 2017, 121, 10467–10478. 10.1021/acs.jpcb.7b09046. 29083167

[ref84] Matsika L. Three-State Conical Intersections in Nucleic Acid Bases. J. Phys. Chem. A 2005, 109, 7538–7545. 10.1021/jp0513622. 16834123

[ref85] Matsika L. Two- and Three-State Conical Intersections in the Uracil Cation. Chem. Phys. 2008, 349, 356–362. 10.1016/j.chemphys.2008.02.027.

[ref86] González-Vázquez J.; González L. Time-Dependent Picture of the Ultrafast Deactivation of keto-Cytosine Including Three-State Conical Intersections. ChemPhysChem 2010, 11, 3617–3624. 10.1002/cphc.201000557. 21069653

[ref87] Pepino A. J.; Segarra-Martí J.; Nenov A.; Rivalta I.; Improta R.; Garavelli M. UV-Induced Long-Lived Decays in Solvated Pyrimidine Nucleosides Resolved at the MS-CASPT2/MM Level. Phys. Chem. Chem. Phys. 2018, 20, 6877–6890. 10.1039/C7CP08235E. 29459916

[ref88] Schwalb N. K.; Temps F. Ultrafast Electronic Relaxation in Guanosine is Promoted by Hydrogen Bonding with Cytidine. J. Am. Chem. Soc. 2007, 129, 9272–9273. 10.1021/ja073448+. 17622153

[ref89] Zhao G.-J.; Han K.-L. Effects of Hydrogen Bonding on Tuning Photochemistry: Concerted Hydrogen-bond Strengthening and Weakening. ChemPhysChem 2008, 9, 1842–1846. 10.1002/cphc.200800371. 18688906

[ref90] Sobolewski A. L.; Domcke W. Computational Studies of the Photophysics of Hydrogen-bonded Molecular Systems. J. Phys. Chem. A 2007, 111, 11725–11735. 10.1021/jp075803o. 17941621

[ref91] El-sayed M. A. Spin-Orbit Coupling and the Radiationless Processes in Nitrogen Heterocyclics. J. Chem. Phys. 1963, 38, 2834–2837. 10.1063/1.1733610.

