# Supplementary material for: Excited-State Properties and Relaxation Pathways of Selenium-Substituted Guanine Nucleobase in Aqueous Solution and DNA Duplex
Source: J Phys Chem B. 2021 Feb 11;125(7):1778–89. doi: 10.1021/acs.jpcb.0c10855 (PMC8023715; doi:10.1021/acs.jpcb.0c10855)
Supplement: Supplementary file 1 — jp0c10855_si_001.pdf [file jp0c10855_si_001.pdf]

Supporting Information

Excited-State Properties and Relaxation Pathways of Selenium-Substituted Guanine Nucleobase in Aqueous Solution and DNA Duplex

Ye-Guang Fang[1#], Danillo Valverde[2#], Sebastian Mai[3,4], Sylvio Canuto[2],

Antonio Carlos Borin[5], Ganglong Cui*[1], Leticia González*[4]

[1]*Key Laboratory of Theoretical and Computational Photochemistry, Ministry of Education, College of Chemistry, Beijing Normal University, Beijing 100875, China*

[2]*Institute of Physics, University of São Paulo, São Paulo, SP, 05508-090, Brazil*

[3]*Photonics Institute, Vienna University of Technology, Gußhausstraße 27-29, 1040 Vienna, Austria*

[4] *Institute of Theoretical Chemistry, Faculty of Chemistry, University of Vienna, Währinger Straße 17, 1090 Vienna, Austria*

[5]*Department of Fundamental Chemistry, Institute of Chemistry, University of São Paulo, Avenida Professor Lineu Prestes, 748, 05508-000, São Paulo, SP, Brazil*

(#equally contribute to the present work)

Email: *ganglong.cui@bnu.edu.cn*; *leticia.gonzalez@univie.ac.at*

Table of Contents

Table of Contents 1

I. Active Spaces 2

II. Superposition of the selected snapshots in water 3

III. Electronic Absorption Spectrum of 6SeG in Water 3

IV. Excited State Minima of 6SeG and 6SeG-C in DNA 4

V. State Intersection Structures 6

VI. Minimum Energy Path in Water 7

VII. Excited State Relaxation Paths 7

VIII. Tables 9

IX. Cartesian Coordinates 11

I. Active Spaces

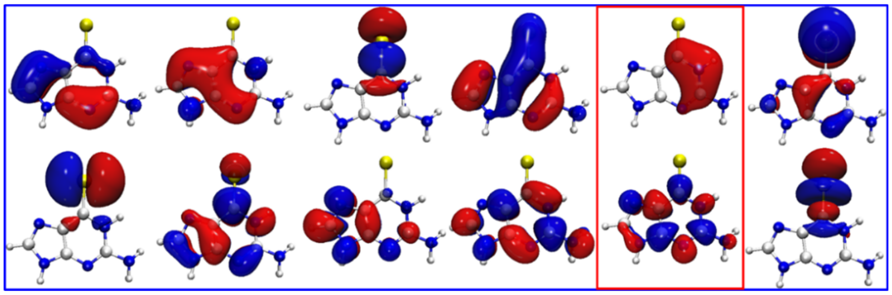

Figure S1. Orbitals included in the active space of 6SeG used in the QM1/MM calculations in the DNA environment. Orbitals in the red frame are excluded in the geometry optimizations. Note that these two orbitals do not correspond to the σ and σ* orbitals located on the C-Se bond (as in Fig. 2 of the main text) but to π,π* orbitals, which were more favourable for the optimization in DNA.

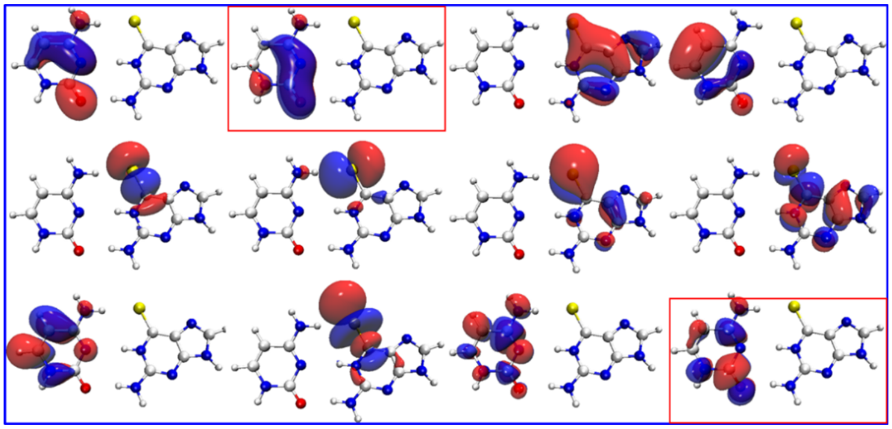

Figure S2. Orbitals included in the active space of the 6SeG-C base pair used for the QM2/MM calculations in the DNA environment. Orbitals in the red frame are excluded in the geometry optimizations.

II. Superposition of the selected snapshots in water

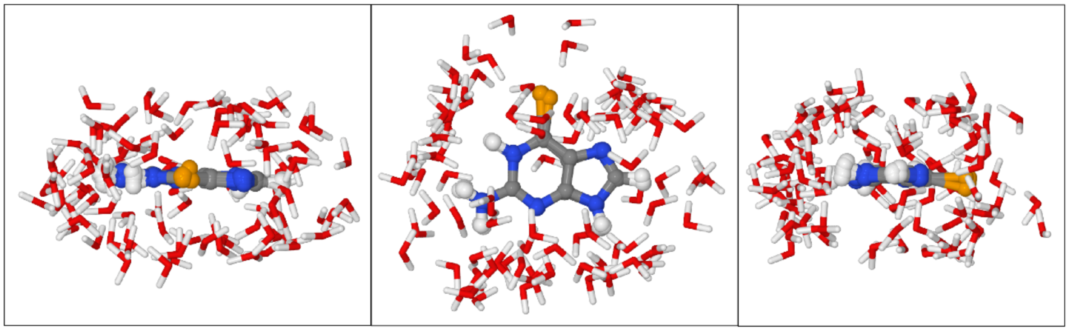

Figure S3. Superposition of the ten selected snapshot in water considering the closest solvent molecules in three viewing angle.

III. Electronic Absorption Spectrum of 6SeG in Water

The electronic absorption spectrum of 6SeG in water solution is based on an ensemble of 500 snapshots taken from a previous classical molecular dynamics simulation, where a Gaussian function (FWHM=0.3 eV) centered on the calculated vertical excitation is superposed, see Figure S3.

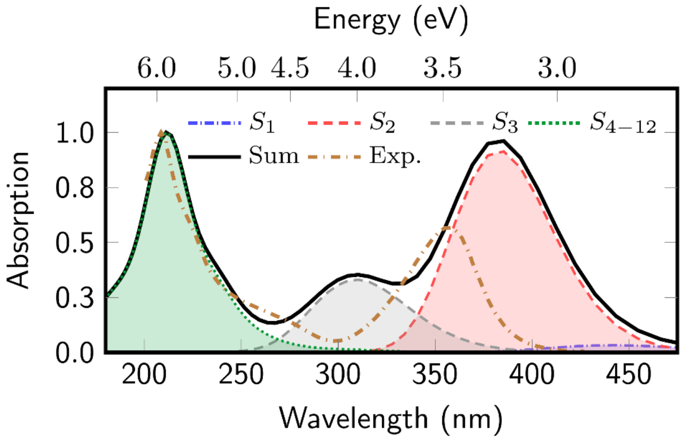

Figure S4. Electronic absorption spectrum of 6SeG in water based on an ensemble of 500 snapshots. At each a vertical QM(MS-CASPT2(14,12))/MM excitation energy calculation is done including 13 singlet states state-averaged.

The spectrum exhibits two well-defined absorption bands with maxima at 3.25 eV (381 nm) and 5.58 eV (222 nm), respectively, in good agreement with the experiment

(357 and 209 nm, respectively). Although the predicted intensity of the bands is at variance with experiment, since the simulated first absorption band is more intense than that reported experimentally, the experimental spectrum is fairly reproduced by our simulations.

Regarding the individual contributions of the electronic states to the absorption spectrum: the first absorption band is best represented by the adiabatic S_2 state, while the adiabatic S_3 state appears with a broad contribution in the range of 250–400 nm. Therefore, we conclude that the S_3 is responsible for the shoulder around 300 nm.

IV.Excited State Minima of 6SeG and 6SeG-C in DNA

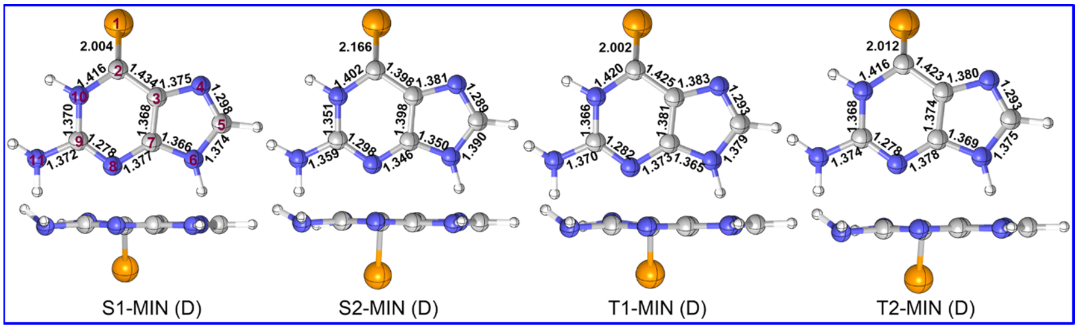

Figure S5. QM(CASSCF)/MM optimized excited-state structures of 6SeG in DNA of "D type", i.e. with the selenium atom in a down position compared to the molecular plane (see also Figure S8).

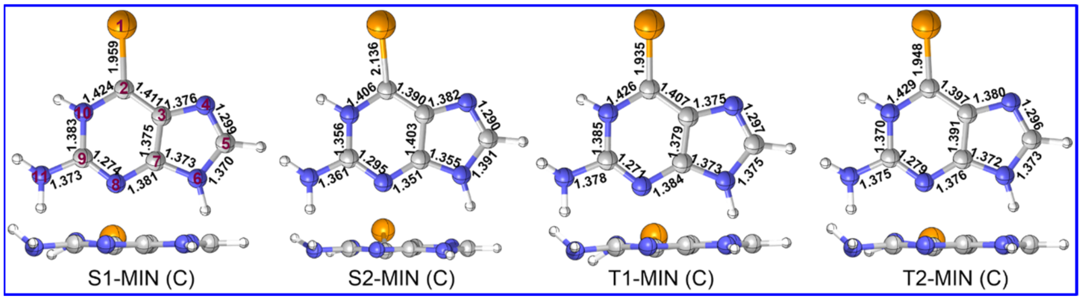

Figure S6. QM(CASSCF)/MM optimized excited-state structures of 6SeG in DNA of "C type", i.e. with the selenium atom lying in the molecular plane, (see also Figure S8).

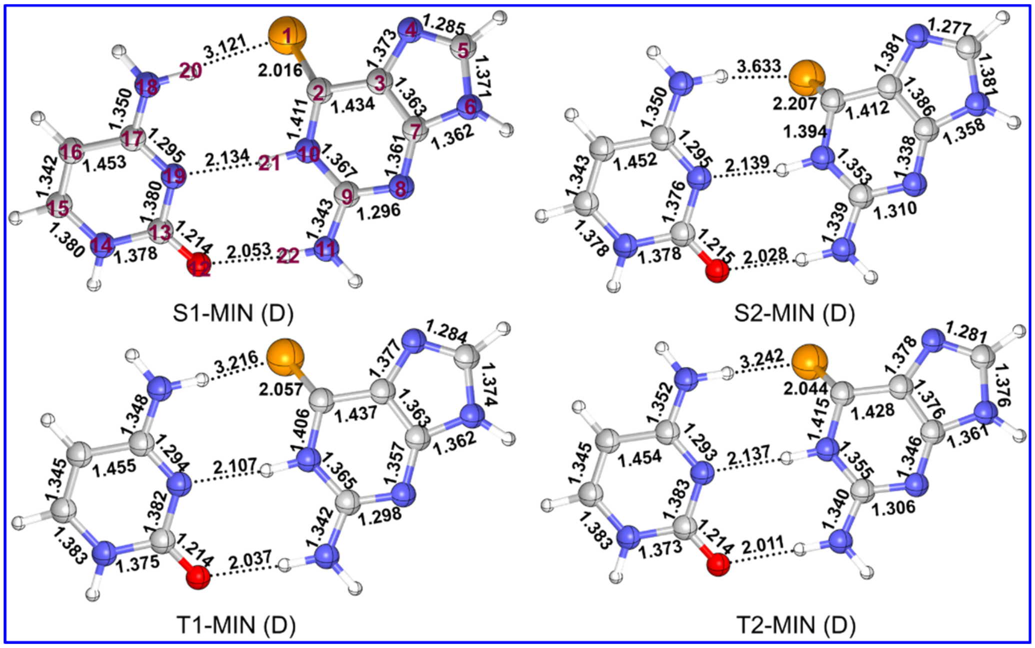

Figure S7. QM(CASSCF)/MM optimized excited-state structures of 6SeG-C in DNA of "D type", i.e. with the selenium atom in a down position compared to the molecular plane.

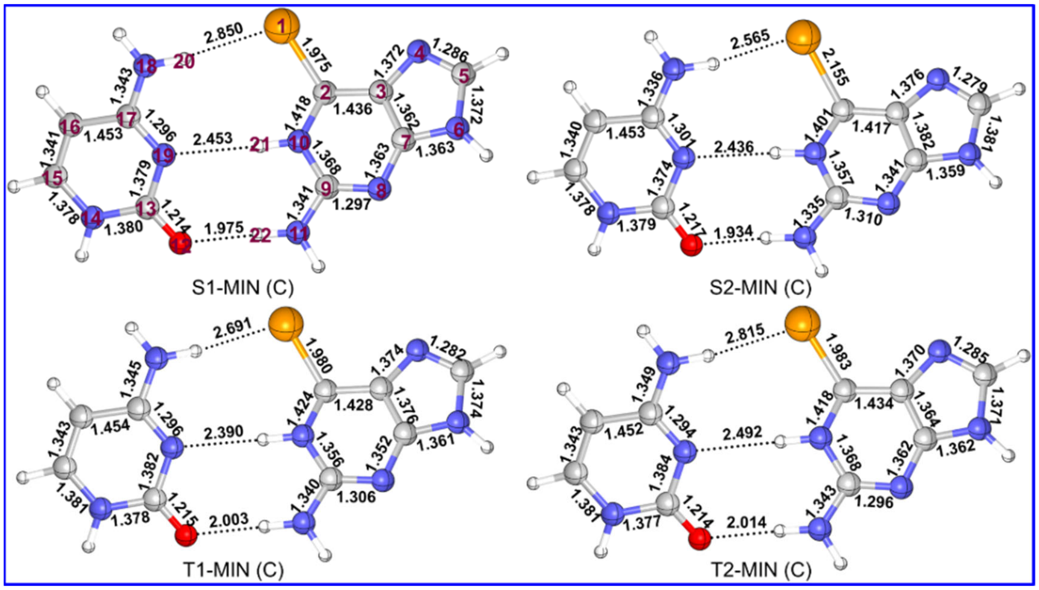

Figure S8. QM(CASSCF)/MM optimized excited-state structures of 6SeG-C in DNA of "C type", i.e. with the selenium atom lying in the molecular plane.

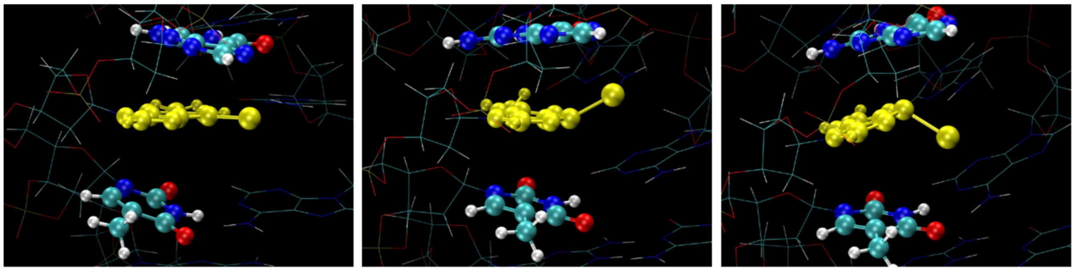

Figure S9. Definition of "C" (left panel), "U" (middle pannel) and "D" (right pannel) conformations of 6SeG (yellow) in DNA, in between Guanine and Thymine, above and below, respectively.

V. State Intersection Structures

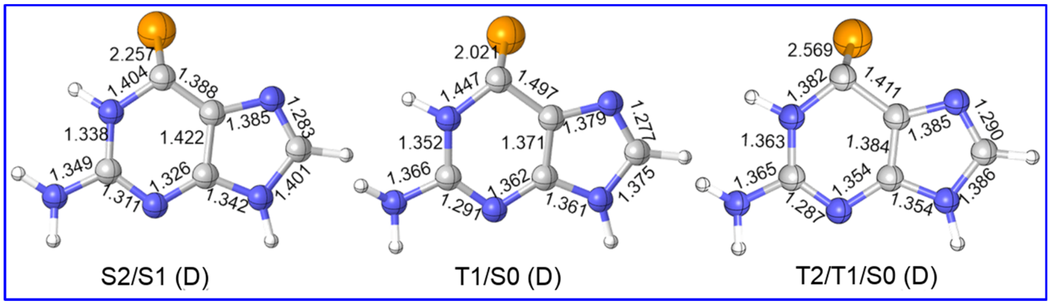

Figure S10. QM(CASSCF)/MM optimized state intersection structures of 6SeG in DNA of "D type", i.e. with the selenium atom in a down position below the molecular plane, (see also Figure S8).

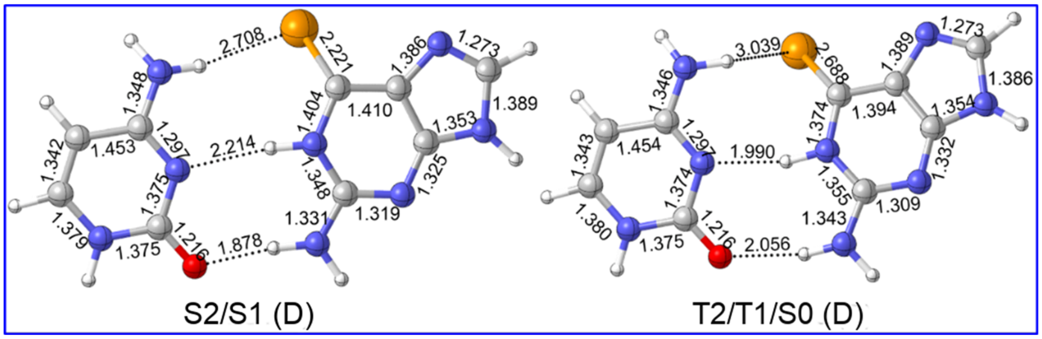

Figure S11. QM(CASSCF)/MM optimized state intersection structures of 6SeG-C in DNA of "D type", i.e. with the selenium atom in a down position below the molecular plane.

VI.Minimum Energy Path in Water

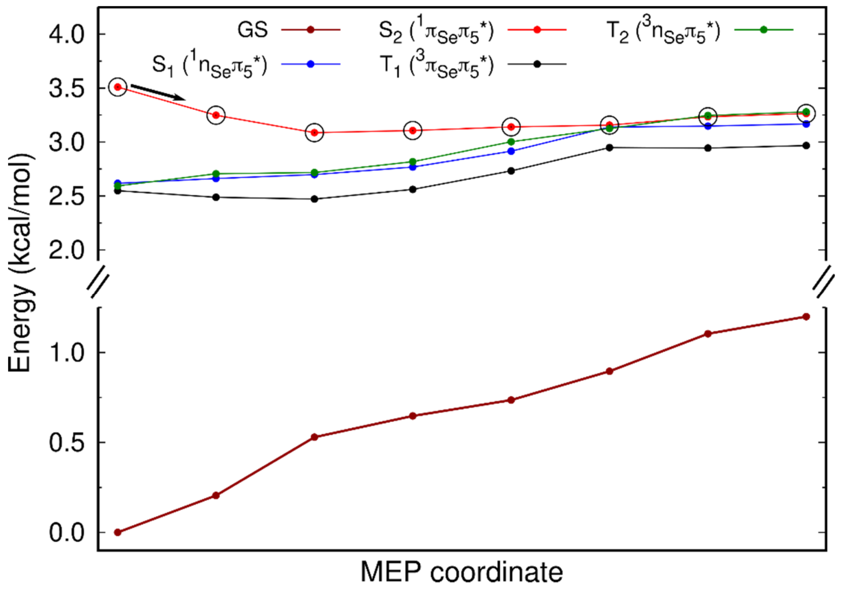

Figure S12. Minimum energy path (MEP) of 6SeG in water along the S_2 ($^1\pi_{Se}\pi_5^*$) state (open circle), from the ground-state optimized geometry, computed at QM(CASSCF(12,9))/MM level. Each point generated was followed by a vertical excitation energy calculation at the QM(MS-CASPT2(14,12))/level of theory.

VII. Excited State Relaxation Paths

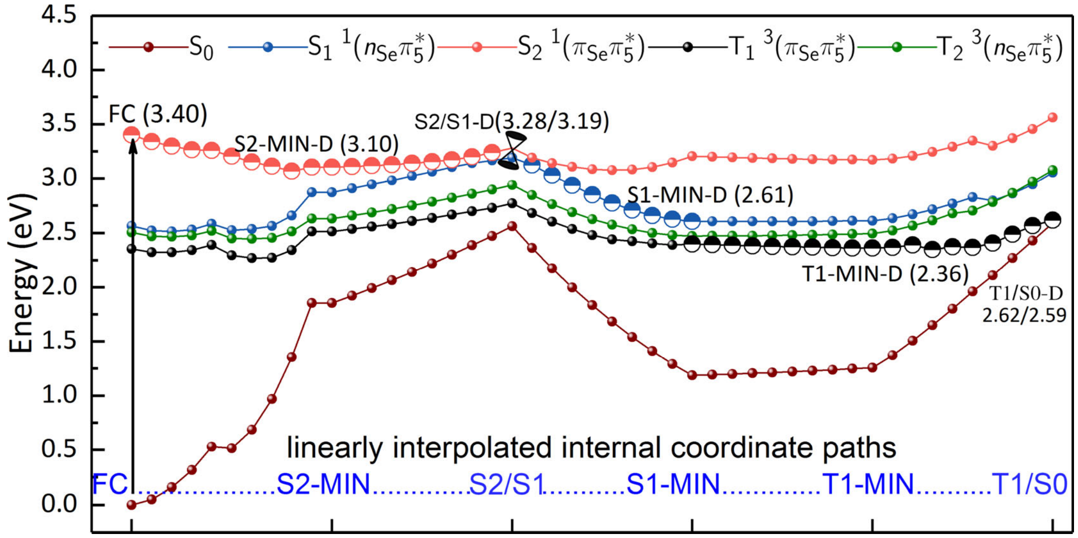

Figure S13. QM(MS-CASPT2)//MM calculated linearly interpolated internal coordinate (LIIC) paths connecting "D type" critical points (minima and intersection structures) of 6SeG. Favorable relaxation pathway is marked with semi-solid cycles.

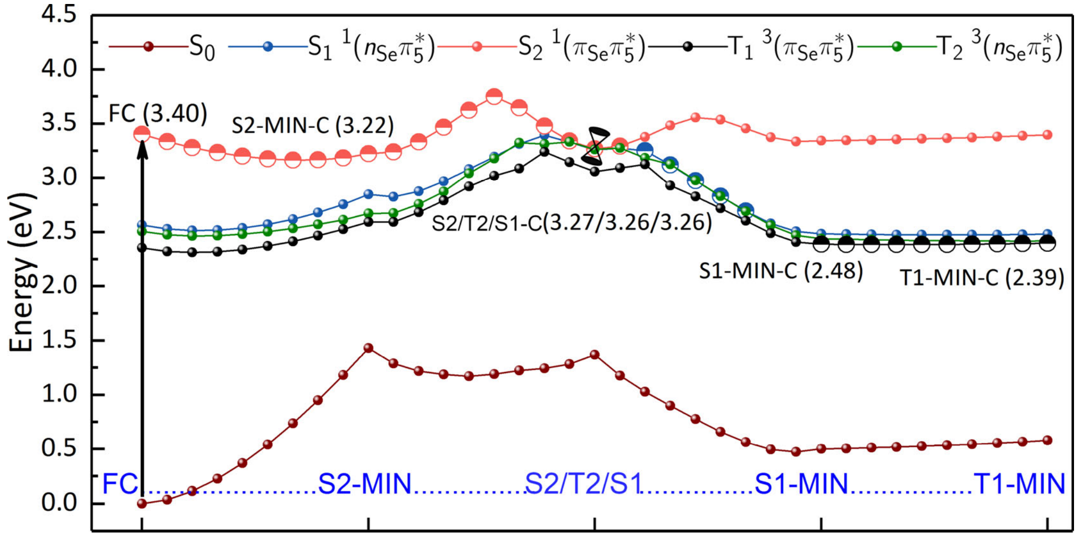

Figure S14. QM(MS-CASPT2)//MM calculated linearly interpolated internal coordinate (LIIC) paths connecting "C type" critical points (minima and intersection structures) of 6SeG. Favorable relaxation pathway is marked with semi-solid cycles.

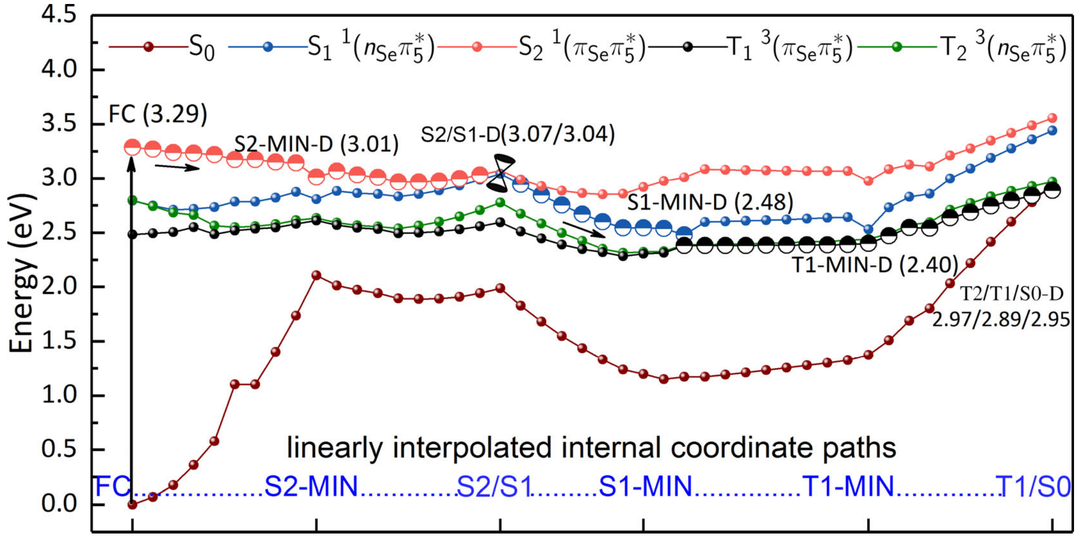

Figure S15. QM(MS-CASPT2)//MM calculated linearly interpolated internal coordinate (LIIC) paths connecting "D type" critical points (minima and intersection structures) of 6SeG-C. Favorable relaxation pathway is marked with semi-solid cycles.

VIII. Tables

Table S1. QM(MS-CASPT2//CASSCF)/MM calculated vertical excitation energies (in eV) of 6SeG in water to the two lowest singlet excited states. Ten snapshots that are randomly sampled from the 1 ns MD simulation are chosen as the starting QM/MM calculations. The calculated root mean square deviations (RMSD) are also given.

Snapshot	S_1 ($^1n\pi^*$)	S_2 ($^1\pi\pi^*$)
1	2.60	3.54
2	2.71	3.53
3	2.77	3.53
4	2.71	3.50
5	2.72	3.55
6	2.79	3.41
7	2.72	3.51
8	2.62	3.56
9	2.67	3.40
10	2.66	3.56
RMSD	0.06	0.06

Table S2. QM(MS-CASPT2//CASSCF)/MM calculated vertical excitation energies (in eV) of 6SeG in DNA to the two lowest singlet excited states. Ten snapshots that are randomly sampled from the 1 ns MD simulation are chosen as the starting QM/MM calculations. The calculated root mean square deviations (RMSD) are also given.

Snapshot	S_1 ($^1n\pi^*$)	S_2 ($^1\pi\pi^*$)
1	2.68	3.39
2	2.70	3.43
3	2.67	3.41
4	2.86	3.35
5	2.79	3.42
6	2.70	3.38
7	2.64	3.45
8	2.62	3.34
9	2.72	3.37
10	2.65	3.40
RMSD	0.07	0.03

Table S3. QM(MS-CASPT2//CASSCF)/MM calculated vertical excitation energies (in eV) of 6SeG-C in DNA to the two lowest singlet excited states. Ten snapshots that are randomly sampled from the 1 ns MD simulation are chosen as the starting QM/MM calculations. The calculated root mean square deviations (RMSD) are also given.

Snapshot	S_1 ($^1n\pi^*$)	S_2 ($^1\pi\pi^*$)
1	2.90	3.38
2	2.79	3.39
3	2.81	3.39
4	2.90	3.36
5	2.87	3.38
6	2.81	3.39
7	2.84	3.40
8	2.84	3.38
9	2.80	3.40
10	2.74	3.37
RMSD	0.05	0.01

Table S4. QM(MS-CASPT2(14,12))/MM calculated energies (in eV) of minima and intersection structures of 6SeG in DNA (labelled as U, D, and C) relative to the S_0 minimum.

	S_1	S_2	T_1	T_2
U	2.46	3.08	2.32	2.35
D	2.61	3.10	2.36	2.48
C	2.48	3.22	2.39	2.43
	T_1/S_0	S_2/S_1	$S_2/S_1/T_2$	$T_2/T_1/S_0$
U	2.62/2.58	3.28/3.20	-	3.04/3.03/2.97
D	2.62/2.59	3.28/3.19	-	3.03/3.01/2.92
C	-	-	3.27/3.26/3.26	-

Table S5. QM(MS-CASPT2(14,12))/MM calculated energies (in eV) of minima and intersection structures of 6SeG-C in DNA (labelled as U, D, and C) relative to the S_0 minimum.

	S_1	S_2	T_1	T_2
U	2.63	2.88	2.29	2.53
D	2.48	3.01	2.40	2.45
C	2.81	3.04	2.36	2.67
	T_1/S_0	S_2/S_1	$S_2/S_1/T_2$	$T_2/T_1/S_0$
U	2.43/2.40	2.90/2.86	-	3.06/2.96/3.03
D	–	3.07/3.04	-	2.97/2.89/2.95
C	–	-	4.11/4.07/4.10	-

Table S6. QM(MS-CASPT2(14,12))/MM calculated five lowest-lying excited states vertical excitation energies (in eV) of 6SeG-C in DNA, along with excited states characters, where LE and CT represent the local excited state and charge transfer state, respectively.

	Character	Energy (eV)
S_1	$^1n\pi^*$ (LE on 6SeG)	2.79
S_2	$^1\pi\pi^*$ (LE on 6SeG)	3.29
S_3	$^1\pi\pi^*$ (LE on Cyt)	4.64
S_4	$^1\pi\sigma^*$ (CT)	4.77
S_5	$^1\pi\pi^*$ (CT)	4.83

IX. Cartesian Coordinates

MS-CASPT2/PCM and QM(CASSCF)/MM optimized minima and intersection structures of 6SeG and 6SeG-C base pair.

```
16
  (S0)min QM(CASSCF)/MM in water
Se  -1.65623547   0.83565008   1.76789592
 C  -0.97597209   0.01721288   0.32815484
 N  -1.67284235  -0.98018992  -0.35585541
 H  -2.66204180  -1.00516026  -0.19283237
 C  -1.21620628  -1.65501339  -1.45660295
 N  -2.12638105  -2.53569491  -1.99862335
 H  -1.72750743  -3.07562820  -2.74124943
 H  -2.59266142  -3.11459543  -1.32651161
 N  -0.04788970  -1.51589173  -1.97124334
 C   0.66257699  -0.49688907  -1.38025265
 N   1.89990332  -0.07234098  -1.72771411
 H   2.41071296  -0.32026755  -2.55006506
 C   2.21423065   0.94678347  -0.86127853
 H   3.14290062   1.48570386  -0.93509235
 N   1.28001521   1.16529119   0.01792216
 C   0.30144785   0.24847091  -0.28050256

  16
  (S1)min QM(CASSCF)/MM in water
Se  -1.70124173   0.68894911   1.97133063
 C  -1.17438345   0.08075143   0.23890364
 N  -1.85415161  -1.05031422  -0.28814920
 H  -2.85552579  -0.96314013  -0.31892342
 C  -1.31906462  -1.78200558  -1.34420640
 N  -2.21737954  -2.71222606  -1.85349036
 H  -1.78510487  -3.26699362  -2.56862707
 H  -2.59670515  -3.30796975  -1.13791949
 N  -0.13974116  -1.65784277  -1.82917696
```

C	0.56720225	-0.60000518	-1.27657598
N	1.84796294	-0.20152930	-1.56743207
H	2.38758707	-0.46487964	-2.36471985
C	2.11081454	0.85562986	-0.74356369
H	3.05333815	1.37521093	-0.77268453
N	1.12422729	1.13815118	0.06308121
C	0.14244766	0.22890622	-0.26255811

16

(S2)min QM(CASSCF)/MM in water

Se	-1.64160682	0.80023964	1.73465732
C	-0.87708105	0.01305029	0.17404151
N	-1.62283057	-0.96562896	-0.42686224
H	-2.59696298	-1.02082660	-0.19905166
C	-1.27754718	-1.51037247	-1.66933077
N	-2.06751605	-2.62441797	-2.01026058
H	-2.07102686	-2.73917444	-3.00882760
H	-1.68876054	-3.47401415	-1.61283330
N	0.07832443	-1.46122571	-2.08564335
C	0.77000613	-0.57320321	-1.47830704
N	2.07644158	-0.19595060	-1.68325671
H	2.58036800	-0.35439028	-2.53208401
C	2.32609458	0.87299240	-0.81928566
H	3.27219897	1.38797898	-0.84547790
N	1.35691324	1.16715599	-0.02572183
C	0.34196108	0.27041936	-0.36302734

16

(T1)min QM(CASSCF)/MM in water

Se	-1.64219400	0.70420616	1.94775923
C	-1.25001680	0.19866567	0.12065176
N	-1.91120465	-0.93808558	-0.40894914
H	-2.91385020	-0.91270366	-0.36333821
C	-1.36731163	-1.73487191	-1.40045415
N	-2.26142062	-2.67060428	-1.89749242
H	-1.80261807	-3.31832970	-2.51016610
H	-2.74922989	-3.16301249	-1.17143995
N	-0.17241792	-1.65577180	-1.84965852
C	0.53422334	-0.59204025	-1.30651394
N	1.82068300	-0.22238208	-1.59337101
H	2.37306744	-0.53548561	-2.36347032
C	2.08508471	0.86737092	-0.81319837
H	3.03271123	1.37653570	-0.85391326
N	1.08466732	1.20381444	-0.04722539
C	0.09687041	0.29203806	-0.34858393

16

(T2)min QM(CASSCF)/MM in water

Se	-1.77877555	0.55291718	2.00138841
C	-1.16041653	0.12259338	0.21267945
N	-1.74634178	-1.04629621	-0.33840769
H	-2.75010039	-1.05523306	-0.32824318
C	-1.16036511	-1.75494433	-1.37236148
N	-1.99181153	-2.71866736	-1.91850155
H	-1.50207452	-3.28812769	-2.58206505
H	-2.43957966	-3.29029893	-1.22555496

N	0.02574483	-1.57388187	-1.82063294
C	0.66609259	-0.49549196	-1.23672236
N	1.92675106	-0.03806076	-1.50902336
H	2.49162969	-0.27989871	-2.29601530
C	2.11878545	1.03996365	-0.69127477
H	3.03431518	1.60603453	-0.70895193
N	1.10232908	1.28571047	0.08636315
C	0.17328711	0.32201772	-0.23973648

16
(S1/S2/T2)CI QM(CASSCF)/MM in water

Se	-1.69135331	0.71396780	1.74490095
C	-0.90966468	-0.01693289	0.16810308
N	-1.63458720	-0.99314143	-0.45275750
H	-2.60197517	-1.09184782	-0.20796719
C	-1.28798011	-1.50658288	-1.69925658
N	-2.06215332	-2.62375906	-2.06185449
H	-2.06386085	-2.72553947	-3.06195761
H	-1.67432489	-3.47367070	-1.67341601
N	0.07413927	-1.43244099	-2.12017582
C	0.74628137	-0.53991457	-1.50070568
N	2.04622409	-0.14115319	-1.69185665
H	2.56001815	-0.28379280	-2.53758572
C	2.27748397	0.91949893	-0.80916539
H	3.21788921	1.44542715	-0.82225494
N	1.29883658	1.18845729	-0.01890356
C	0.29830622	0.28439141	-0.37589451

16
(S0/T1)ISC QM(CASSCF)/MM in water

Se	-1.17049900	0.43224970	2.02880006
N	-1.31379238	0.36754263	0.01958306
C	-1.91074454	-0.85168534	-0.45748208
N	-2.91348138	-0.85200510	-0.40759454
C	-1.34684953	-1.67061892	-1.40795611
N	-2.22387064	-2.61808602	-1.90288750
C	-1.74530386	-3.32275609	-2.43166817
N	-2.80275906	-3.03068475	-1.19470421
N	-0.14335313	-1.63229709	-1.81228238
C	0.56540426	-0.56510481	-1.24386408
C	1.84343714	-0.19747048	-1.52039351
H	2.42126107	-0.54999623	-2.25966034
H	2.09607375	0.91541302	-0.77387433
H	3.04660794	1.41409781	-0.80303576
H	1.07351428	1.28328999	-0.04240478
H	0.09514789	0.35674038	-0.33316121

16
(S0)min MS-CASPT2 PCM in water

Se	2.41183838	-0.09566420	0.00699822
N	-2.49990843	-1.58905520	-0.00097685
C	-1.59050630	-2.62536874	0.00037770
N	-0.32502682	-2.23193438	0.00144987
C	-1.76889763	-0.42324567	-0.00031184
N	-2.26379786	0.83446966	-0.00411308
C	-1.29417583	1.74912199	-0.00493140
N	-1.60989826	3.07539737	0.08111611

N	0.04472038	1.43244759	-0.01227227
C	0.58445508	0.15901636	-0.00335003
C	-0.42042661	-0.83965547	0.00079979
H	-3.52669876	-1.66993728	-0.00012296
H	-2.57690820	3.27660515	-0.19579132
H	-0.92680831	3.72983472	-0.31892926
H	0.71660648	2.21811596	0.00135918
H	-1.92644629	-3.66567385	0.00024514

16
(S1)min MSCASPT2 PCM in water

Se	-2.31753320	-0.20768906	0.50821673
N	2.50856707	-1.56486446	0.04141661
C	1.62675553	-2.62002489	-0.01853782
N	0.35223086	-2.21878405	-0.13133493
C	1.77425513	-0.41033804	-0.02725970
N	2.26136017	0.87049907	0.03527847
C	1.27295797	1.76751410	-0.01859266
N	1.58641553	3.10795444	-0.06990277
N	-0.06355227	1.46223492	-0.10532396
C	-0.57917643	0.15378010	-0.27669883
C	0.43934374	-0.83992670	-0.14218165
H	3.52986275	-1.62945970	0.14056621
H	2.53182529	3.28697320	0.28588640
H	0.89203296	3.72477844	0.36842602
H	-0.70510709	2.25451000	-0.25390591
H	1.96804321	-3.65577711	0.02340783

16
(S2)min MSCASPT2 PCM in water

Se	-2.23868634	-0.09267259	0.81108800
N	2.50219763	-1.56855552	0.09039633
C	1.63369563	-2.62109237	-0.06725246
N	0.37169648	-2.23582193	-0.25213020
C	1.75855132	-0.40784540	0.00110336
N	2.23821053	0.85102062	0.08724782
C	1.26840139	1.76351963	-0.04251969
N	1.59698749	3.09018364	-0.04125699
N	-0.05081154	1.44165542	-0.23668820
C	-0.58482074	0.14932847	-0.36074384
C	0.44214693	-0.84309179	-0.20857421
H	3.52113255	-1.63035962	0.25755860
H	2.50319477	3.27793645	0.40038683
H	0.85623120	3.73915218	0.24928571
H	-0.72336967	2.22371597	-0.29280753
H	1.98352360	-3.65569291	-0.03563348

16
(T1)min MSCASPT2 PCM in water

Se	-2.24406027	-0.16038551	0.70182762
N	2.51067146	-1.56083057	0.07179285
C	1.63042599	-2.61401772	-0.05681381
N	0.36282514	-2.22538462	-0.20657832
C	1.76813457	-0.40928522	0.01137688
N	2.24094735	0.85328652	0.08909173
C	1.25365916	1.76597600	-0.02323201
N	1.60301699	3.08701965	-0.04521784

N	-0.05708196	1.45366848	-0.18831698
C	-0.60777240	0.14844802	-0.33535192
C	0.42624681	-0.84690759	-0.16692938
H	3.52816417	-1.62373212	0.21007711
H	2.52319276	3.26657507	0.36948130
H	0.87982600	3.75500607	0.24438788
H	-0.71541584	2.24202893	-0.28062991
H	1.97550131	-3.65008517	-0.03550517

16
(T2)min MSCASPT2 PCM in water

Se	-2.27194698	-0.16161588	0.73496930
N	2.50788385	-1.56562420	0.07176403
C	1.62399502	-2.61868769	-0.04691686
N	0.36343451	-2.21544884	-0.21806146
C	1.76813457	-0.40928522	0.01137688
N	2.24094735	0.85328652	0.08909173
C	1.25365916	1.76597600	-0.02323201
N	1.58550070	3.09608414	-0.05185199
N	-0.06023812	1.44878619	-0.19507242
C	-0.58773456	0.15605078	-0.33324045
C	0.45437332	-0.83850837	-0.20679703
H	3.52415611	-1.63331101	0.21791710
H	2.51747235	3.28474798	0.33364129
H	0.86675627	3.72529980	0.32533778
H	-0.70477783	2.24465023	-0.32229180
H	1.96310544	-3.65542245	-0.00567374

16
S0-MIN-C (6SeGua in DNA)

N	29.88195123	29.49685739	27.85211766
C	30.83514336	28.49217539	27.89653674
N	30.35309603	27.34658971	28.24296027
C	29.01322211	27.58330471	28.44559882
C	27.97258753	26.69042893	28.80701699
Se	28.07553350	24.89618840	29.13639390
N	26.75361532	27.36810067	28.89157707
C	26.56566729	28.69162330	28.64311795
N	25.29804532	29.15869197	28.83771330
N	27.49508886	29.50965598	28.28886966
C	28.71283726	28.91235760	28.21029786
H	31.86812679	28.68419375	27.67098066
H	25.97616671	26.82393488	29.20491991
H	24.55076857	28.53035627	28.61887259
H	25.16723115	30.07689421	28.46155253
H	30.01405605	30.45834384	27.62435408

16
S1-MIN-C (6SeGua in DNA)

N	29.85804342	29.50057885	27.84510034
C	30.78870343	28.50326152	27.97507734
N	30.29591925	27.38472196	28.41451654
C	28.95754499	27.65057704	28.59351462
C	27.91779597	26.83408368	29.08642454
Se	28.00040951	24.90791538	29.43192286
N	26.62951679	27.43078545	28.97921558

C	26.49597304	28.77045426	28.66447174
N	25.22097204	29.24988466	28.83618755
N	27.42331849	29.54993565	28.26937990
C	28.66676090	28.94856432	28.24602397
H	31.82453927	28.65737277	27.72970001
H	25.95406058	27.11734045	29.64808621
H	24.49161549	28.61904127	28.56443611
H	25.10856571	30.16100368	28.43621913
H	30.02184414	30.45200260	27.60176126

16

S2-MIN-C (6SeGua in DNA)

N	29.86942503	29.49239368	27.84579449
C	30.83708625	28.50989216	28.03438001
N	30.37819232	27.41885928	28.54439784
C	29.02749406	27.65139201	28.70672716
C	28.04254755	26.90604060	29.35164107
Se	28.00765293	24.74952027	29.21912330
N	26.76911395	27.47435248	29.19433964
C	26.55080241	28.72143951	28.71308299
N	25.27103557	29.18133731	28.78788092
N	27.47611705	29.50229090	28.25098159
C	28.70302724	28.93910692	28.25641189
H	31.86537932	28.67887196	27.76894675
H	26.02312602	27.01219405	29.67298224
H	24.54370102	28.50033104	28.69804025
H	25.11663692	30.01184124	28.25181807
H	30.01332736	30.44353219	27.58237269

16

T1-MIN-C (6SeGua in DNA)

N	29.85015114	29.49879319	27.84685364
C	30.77664816	28.49916325	28.03072850
N	30.26914342	27.40289354	28.50356304
C	28.93114217	27.67960086	28.65450311
C	27.89685716	26.91514521	29.22530801
Se	27.98645445	25.00685878	29.53427402
N	26.60760317	27.50865084	29.08822997
C	26.47855627	28.82555923	28.67843588
N	25.19718056	29.31218605	28.81904531
N	27.40583840	29.57502647	28.23715389
C	28.64944553	28.96774833	28.25014236
H	31.81593042	28.63717944	27.78892089
H	25.96013793	27.27823144	29.81688862
H	24.48512940	28.67537723	28.51310213
H	25.10533535	30.20817245	28.37978254
H	30.01625536	30.44337983	27.59080419

16

T2-MIN-C (6SeGua in DNA)

N	29.85106539	29.49543589	27.84925785
C	30.77866749	28.49547617	28.01027350
N	30.28551819	27.38594780	28.46450508
C	28.94062484	27.64651539	28.62783493
C	27.91018286	26.87031288	29.16310098
Se	28.01250116	24.97579567	29.60364292
N	26.62271306	27.47761227	29.03304684

C	26.48580298	28.79631859	28.68909480
N	25.21464296	29.29845393	28.84289274
N	27.42153919	29.56232809	28.27262103
C	28.65348542	28.95126065	28.23953856
H	31.81631565	28.64656493	27.76813284
H	25.89974003	27.09650985	29.60687790
H	24.48531898	28.67687045	28.54813913
H	25.13007643	30.20102190	28.41636911
H	30.01621046	30.43893496	27.59540601

16

S2S1T2 (6SeGua in DNA)

N	29.83298957	29.51139276	27.85941445
C	30.74329164	28.46310481	27.93255604
N	30.25278065	27.33237828	28.24858411
C	28.87228223	27.59987468	28.42078971
C	27.85903023	26.83445237	28.84960312
Se	28.07630820	24.93615421	29.23952042
N	26.64636128	27.42758500	28.96186272
C	26.38368926	28.71643302	28.46635945
N	25.17247424	29.24035966	28.97094194
N	27.45529727	29.57633722	28.24241717
C	28.59911785	28.98366055	28.17184836
H	31.78837196	28.63006331	27.72674312
H	25.83885443	26.90390036	29.21848880
H	24.75396825	29.83732961	28.27948257
H	25.35282336	29.82539499	29.77491486
H	30.02458943	30.45823078	27.61888733

16

S1-MIN-U (6SeGua in DNA)

N	29.84064080	29.51235070	27.84777609
C	30.73969478	28.47833899	27.90109555
N	30.20724207	27.35617902	28.28045251
C	28.88302119	27.66160575	28.49382518
C	27.80300112	26.83106456	28.90938961
Se	27.69119519	24.95597498	28.27828515
N	26.58065539	27.54798226	28.96534455
C	26.47001902	28.87596059	28.64564662
N	25.20646555	29.38856656	28.82626053
N	27.42109734	29.62926145	28.25120785
C	28.63895924	28.98363213	28.22795976
H	31.77920962	28.61550703	27.66278854
H	25.85448439	27.18368316	29.54657249
H	24.47303101	28.80557617	28.46831785
H	25.14077900	30.32459792	28.47495577
H	30.01193982	30.45454718	27.56758618

16

S2-MIN-U (6SeGua in DNA)

N	29.83815943	29.50511185	27.85599359
C	30.76681110	28.48531330	28.02856180
N	30.26156400	27.39360818	28.49025092
C	28.91220871	27.67107424	28.64303507
C	27.86835374	26.88798245	29.15652420
Se	27.68999782	24.91874113	28.30740842
N	26.64371983	27.56040970	29.07621998

C	26.48131060	28.83539016	28.66611971
N	25.22162851	29.35101357	28.74593634
N	27.44854206	29.59189903	28.24744443
C	28.64554772	28.98229164	28.24883828
H	31.80516168	28.62587223	27.78454368
H	25.84721860	27.07239146	29.43254629
H	24.46545887	28.71352410	28.59281351
H	25.12354531	30.21251329	28.24606423
H	30.01387204	30.44189387	27.56830293

16

T1-MIN-U (6SeGua in DNA)

N	29.82506165	29.51641611	27.84484262
C	30.71051599	28.46575552	27.88961137
N	30.17682450	27.34711036	28.25028342
C	28.84442303	27.65478754	28.47050624
C	27.77504559	26.83578450	28.91469448
Se	27.69948331	24.97535482	28.27418978
N	26.56293922	27.56405872	28.99168205
C	26.44274026	28.88769034	28.64557286
N	25.17266171	29.39352287	28.83395523
N	27.38625848	29.63609996	28.23741673
C	28.60821869	28.99291826	28.21672464
H	31.75275541	28.59627374	27.65561794
H	25.82562553	27.19360562	29.55288718
H	24.44924796	28.81237200	28.45258022
H	25.10681794	30.32860542	28.47942972
H	30.01214026	30.45726887	27.57096576

16

T2-MIN-U (6SeGua in DNA)

N	29.84989810	29.50684638	27.84694862
C	30.75813725	28.47828100	27.91114058
N	30.24495884	27.35312809	28.28961055
C	28.90881204	27.63502044	28.49759784
C	27.85020343	26.80947137	28.94102247
Se	27.69198756	24.95002322	28.28142732
N	26.61858829	27.52022596	28.96415984
C	26.48637625	28.84092174	28.64931406
N	25.21783975	29.34609385	28.81263466
N	27.43511472	29.60489727	28.26110158
C	28.65269450	28.97000257	28.23332948
H	31.79656897	28.62600109	27.67212154
H	25.86115012	27.08580630	29.44793193
H	24.48470183	28.74215131	28.49277235
H	25.13924885	30.26552023	28.42301242
H	30.01212117	30.44901808	27.56899943

16

S2S1-U (6SeGua in DNA)

N	29.83754714	29.50962012	27.85311800
C	30.76042021	28.46028805	27.92344201
N	30.23763017	27.33435096	28.25120803
C	28.88919007	27.60372198	28.41203604
C	27.84077400	26.79615792	28.83646507
Se	27.40190297	25.30442382	27.19570496
N	26.63590191	27.51316197	28.84237707

C	26.49246490	28.82508807	28.61332705
N	25.25649481	29.35768411	28.74462406
N	27.48447197	29.61488013	28.28743003
C	28.64599006	28.98145208	28.17747902
H	31.80082229	28.62344805	27.70506599
H	25.82084685	26.93867593	28.92852508
H	24.46586676	28.74872806	28.70731306
H	25.13575380	30.25615317	28.32446104
H	30.01301515	30.46110019	27.60837698

16
T2T1S0-U (6SeGua in DNA)

N	29.82097729	29.51401310	27.85572992
C	30.72252108	28.46075446	27.95659809
N	30.17915679	27.35508912	28.33308679
C	28.83769981	27.66306610	28.50203725
C	27.77319385	26.84194978	28.89991472
Se	27.30570326	24.50964724	27.78877528
N	26.61033224	27.57894237	28.93991071
C	26.46390054	28.89259609	28.62293743
N	25.21491568	29.41962866	28.77803730
N	27.43729577	29.65070785	28.24266371
C	28.61989332	29.00373480	28.20544378
H	31.76604846	28.59817998	27.73324850
H	25.79119214	27.08676054	29.23454626
H	24.44367798	28.80883503	28.59543303
H	25.11591234	30.32281147	28.35830637
H	30.01092174	30.45431060	27.58306104

16
T1S0-U (6SeGua in DNA)

N	29.82090561	29.51179695	27.85296411
C	30.72397985	28.48239320	27.99056308
N	30.18038693	27.37869648	28.33724982
C	28.83462421	27.66468790	28.43916224
C	27.70627163	26.79377218	28.89202904
Se	27.66226790	25.63153993	27.24731602
N	26.54177973	27.64202394	28.97028972
C	26.45022482	28.94264988	28.61820499
N	25.22730903	29.52129673	28.80344741
N	27.42538582	29.66904523	28.18259554
C	28.60164434	28.98414022	28.14560636
H	31.77506894	28.62445473	27.80497967
H	25.74153647	27.23857200	29.40763736
H	24.42832452	28.94240931	28.64024119
H	25.16084965	30.42729342	28.38449691
H	30.00646940	30.46061560	27.61142422

16
S1-MIN-D (6SeGua in DNA)

N	29.82402524	29.50804943	27.82581813
C	30.73759797	28.50288034	28.03163536
N	30.21411803	27.43653873	28.55415964
C	28.87790055	27.73689720	28.67457407
C	27.80863035	26.96611039	29.23802265
Se	28.14608768	26.20014136	31.05907958
N	26.56858860	27.62532348	29.05434855

C	26.44927128	28.92597129	28.64225654
N	25.18161894	29.44217335	28.74140405
N	27.40648145	29.65616705	28.21365947
C	28.62010033	29.00501593	28.23147256
H	31.77812697	28.61931478	27.78462851
H	25.75731674	27.20818501	29.45658903
H	24.44052783	28.79917086	28.54013514
H	25.08722601	30.29900968	28.23161533
H	29.97994807	30.40438857	27.41615055

16

S2-MIN-D (6SeGua in DNA)

N	29.81571465	29.51062899	27.83161044
C	30.72797174	28.46856073	27.95315147
N	30.20547079	27.38098101	28.40658178
C	28.86815441	27.68123537	28.57734235
C	27.81003982	26.89763760	29.04676239
Se	28.14749354	26.18976983	31.06565171
N	26.60003106	27.60555804	29.01879316
C	26.45768554	28.88889621	28.62193184
N	25.22041987	29.43562692	28.75309401
N	27.43071963	29.63375513	28.19485558
C	28.62117481	29.00614130	28.20737585
H	31.76711097	28.60364349	27.71096950
H	25.82484445	27.15924407	29.46193524
H	24.43036749	28.82924006	28.66831683
H	25.12209982	30.31343320	28.28407204
H	29.96261226	30.40956137	27.42573266

16

T1-MIN-D (6SeGua in DNA)

N	29.81716462	29.50908994	27.84883279
C	30.72735022	28.49420317	28.05519777
N	30.21075868	27.43661352	28.59123543
C	28.86826388	27.73956343	28.73093575
C	27.79025201	26.95440641	29.23332944
Se	28.15087264	26.20751254	31.05534165
N	26.55333161	27.62505195	29.03851903
C	26.44083622	28.92883913	28.64822497
N	25.17820910	29.45222616	28.74000018
N	27.40631034	29.66600348	28.23891682
C	28.61612638	29.01758378	28.27135444
H	31.76555086	28.60475933	27.79436247
H	25.74878374	27.22285249	29.46911398
H	24.42786222	28.81786035	28.54848450
H	25.08981747	30.31648246	28.24255635
H	29.97220619	30.39567883	27.42162915

16

T2-MIN-D (6SeGua in DNA)

N	29.82791758	29.50757124	27.82495303
C	30.74658519	28.50095387	28.00518586
N	30.24006972	27.42253678	28.50856702
C	28.89741224	27.70975690	28.64492554
C	27.85260888	26.92682051	29.21005187
Se	28.14654754	26.19681428	31.06170268
N	26.60983129	27.59563830	29.09669839

C	26.46176181	28.88996309	28.67836230
N	25.19077132	29.39574258	28.80437749
N	27.40382285	29.62578395	28.22608092
C	28.62453578	28.98651441	28.21759100
H	31.78439880	28.63357111	27.75329843
H	25.82351392	27.13557542	29.50475112
H	24.45048240	28.75030955	28.60786882
H	25.08483573	30.25808549	28.30645787
H	29.97559174	30.40371008	27.41744728

16
S2S1-D (6SeGua in DNA)

N	29.82329614	29.51425612	27.86011100
C	30.73645821	28.45470605	27.93949601
N	30.20651317	27.34301696	28.30045203
C	28.86666107	27.63415898	28.49895705
C	27.78330500	26.81082292	28.77372606
Se	27.99530201	26.04621987	30.88710222
N	26.60812291	27.56849698	28.90264308
C	26.47185390	28.86998807	28.62247106
N	25.26047582	29.43164812	28.81445007
N	27.46293197	29.63329613	28.22863403
C	28.63185406	29.00694508	28.21171203
H	31.77912928	28.60831905	27.72414199
H	25.86326486	27.09141795	29.37192311
H	24.44325975	28.85960107	28.80628207
H	25.16077681	30.37189418	28.49430305
H	30.01196816	30.46654619	27.62790699

16
T2T1S0-D (6SeGua in DNA)

N	29.82195180	29.51521538	27.85110627
C	30.72068329	28.46548102	27.95464167
N	30.17385843	27.35884307	28.33038158
C	28.83423583	27.66986458	28.49440051
C	27.76261008	26.83753229	28.88055198
Se	28.24360646	25.42124842	30.96981743
N	26.60067500	27.58363005	28.92557147
C	26.46261385	28.90519843	28.62110386
N	25.21420324	29.43407834	28.78279978
N	27.43413477	29.66034455	28.24241109
C	28.61872835	29.00553917	28.20476563
H	31.76587539	28.60088483	27.73802097
H	25.77666377	27.10194831	29.22085966
H	24.44056572	28.82861856	28.59378268
H	25.11938356	30.34098371	28.37046880
H	30.01212023	30.45442460	27.57582190

16
T1S0-D (6SeGua in DNA)

N	29.84368634	29.50810638	27.85463774
C	30.75028049	28.47389645	27.86867387
N	30.23033243	27.35465865	28.19835619
C	28.90151607	27.63738157	28.43547656
C	27.77329415	26.70057026	28.73871751
Se	28.35478473	26.26472115	30.62407476
N	26.57361171	27.50871138	28.74689174

C	26.48168158	28.84704217	28.58137857
N	25.24154479	29.38775854	28.76687128
N	27.46422953	29.63391230	28.29374782
C	28.65151489	28.96992880	28.23059562
H	31.78817241	28.63115617	27.63317775
H	25.73831595	27.03743193	29.01873062
H	24.45867366	28.81485660	28.52327141
H	25.17374304	30.32673622	28.42762654
H	30.01088139	30.46503599	27.63257309

29
S0-MIN-C (6SeG-Cyt in DNA)

N	29.96042089	29.06936119	28.38069983
C	30.78976812	27.96942208	28.48687432
N	30.16427193	26.88164829	28.70252561
C	28.83640647	27.24619507	28.75270825
C	27.67786542	26.46535458	28.90938852
Se	27.61789125	24.63229816	29.13805382
N	26.52196159	27.23937785	28.88486829
C	26.49186172	28.58523236	28.69763192
N	25.29608229	29.17468820	28.70505847
N	27.56461160	29.31840459	28.51589806
C	28.69557143	28.60745717	28.55092416
H	31.85802127	28.06378842	28.38064357
H	25.63857017	26.76644723	28.96256968
H	24.42691239	28.69486655	28.86261702
H	25.28810303	30.16386462	28.58694535
N	21.10621649	26.38079551	28.90480468
C	22.39224499	26.88281219	28.88002549
O	22.61224462	28.06377329	29.07721070
N	23.42990503	26.01723329	28.62548866
C	23.20685748	24.74610130	28.45170563
N	24.22300748	23.92273928	28.19093814
C	21.87219539	24.17599006	28.53338149
C	20.86647745	25.03012496	28.76707099
H	25.17257861	24.24763001	28.23400746
H	24.07061728	22.94206513	28.11699782
H	21.70143674	23.11759462	28.43242135
H	19.84649753	24.69771661	28.86738604
H	30.23813733	30.00628412	28.19261536
H	20.36263966	27.02061316	29.10005894

29
S1-MIN-C (6SeG-Cyt in DNA)

N	30.00863860	29.09345770	28.36438021
C	30.83856828	28.02306596	28.58193530
N	30.19831625	26.95825987	28.91388582
C	28.87789178	27.33246398	28.92333366
C	27.69264943	26.61819937	29.30576849
Se	27.62559749	24.64762129	29.19438644
N	26.51453001	27.33102588	28.96888217
C	26.52459812	28.66904302	28.68578939
N	25.32314016	29.25756612	28.59149492
N	27.59422144	29.37514021	28.48610245
C	28.74035886	28.64504896	28.58590194
H	31.90540760	28.09640380	28.45524193
H	25.62276396	26.92738138	29.17496999
H	24.46243764	28.79679176	28.81272118
H	25.32220783	30.23857900	28.42181108
N	21.15948899	26.31175392	28.89838970
C	22.45074389	26.79664994	28.86586967
O	22.69067922	27.96686925	29.08290653
N	23.47024877	25.91526788	28.57491085

C	23.22107432	24.65522069	28.39884049
N	24.21879511	23.82765108	28.04786661
C	21.89138059	24.09133722	28.55573484
C	20.90105363	24.96173734	28.80009818
H	25.13405391	24.19186678	27.88811933
H	24.07712214	22.84488227	27.98852734
H	21.70607428	23.03391300	28.47281713
H	19.87955962	24.64564656	28.93100886
H	30.25841550	30.03795461	28.16433626
H	20.43509548	26.96947867	29.10207351

29

S2-MIN-C (6SeG-Cyt in DNA)

N	29.99932319	29.14550499	28.44128016
C	30.84745620	28.14385857	28.86945910
N	30.23075168	27.10478952	29.28781759
C	28.89573143	27.40048470	29.13689812
C	27.73265430	26.71264650	29.56190523
Se	27.64078513	24.59407513	29.17923200
N	26.56197957	27.36106979	29.14652605
C	26.52515664	28.61165766	28.61990525
N	25.32054277	29.12437333	28.35826478
N	27.59444750	29.32252247	28.36259943
C	28.73853046	28.66968703	28.61378912
H	31.91799290	28.24967714	28.81634272
H	25.68405350	26.90187068	29.30634080
H	24.46417783	28.70612238	28.67522961
H	25.30247105	30.06099449	28.01939422
N	21.19592265	26.34600388	28.93630964
C	22.48471105	26.83628903	28.93054175
O	22.70906427	28.01947190	29.10766822
N	23.51784852	25.95597050	28.71675377
C	23.27994140	24.68976530	28.53916040
N	24.29222292	23.85824208	28.27523562
C	21.94057894	24.13071213	28.60482902
C	20.94076625	24.99712119	28.81474415
H	25.23503484	24.18148949	28.39036594
H	24.14190758	22.87398859	28.29429372
H	21.76217573	23.07271854	28.51332578
H	19.91603879	24.67787134	28.90784506
H	30.23803448	30.06849712	28.14931983
H	20.46280479	27.00138801	29.10557900

29

T1-MIN-C (6SeG-Cyt in DNA)

N	29.99876297	29.08809368	28.39618886
C	30.82137470	28.01997376	28.65951331
N	30.17715315	26.96311907	28.99540604
C	28.85260282	27.32713548	28.95690765
C	27.66578272	26.63609794	29.34802738
Se	27.65461830	24.66428339	29.16849487
N	26.50316871	27.34397589	28.92993302
C	26.51080385	28.66658289	28.63172570
N	25.31956641	29.26522305	28.49317269
N	27.59422958	29.37266041	28.45157066
C	28.72571814	28.64522487	28.58326919
H	31.89167099	28.09386135	28.56214258
H	25.61042011	26.90780642	29.06155704
H	24.45802883	28.83047114	28.76184102
H	25.33946273	30.24897258	28.33859549
N	21.14960914	26.32328395	28.90839172
C	22.43750405	26.81326193	28.89772520
O	22.67247059	27.98762272	29.10005770
N	23.46940252	25.92959321	28.64689527

C	23.23330124	24.66691463	28.47216710
N	24.24200264	23.83397142	28.16074976
C	21.89708378	24.10281028	28.57309563
C	20.89642414	24.97037250	28.79371403
H	25.18462665	24.16909667	28.22207167
H	24.10671023	22.85131011	28.25427332
H	21.71637052	23.04575683	28.47737429
H	19.87324651	24.64989850	28.89607633
H	30.26444651	30.01900577	28.16971567
H	20.41209171	26.97013949	29.10027397

29

T2-MIN-C (6SeG-Cyt in DNA)

N	30.03823661	29.11654168	28.39909653
C	30.87165500	28.05769018	28.65129523
N	30.23376992	26.98946636	28.97372380
C	28.91140983	27.34644417	28.93468551
C	27.72696680	26.63024193	29.31088502
Se	27.65204857	24.65218474	29.18932225
N	26.55678906	27.31104239	28.88957122
C	26.55222371	28.64529890	28.58783899
N	25.34487289	29.21229263	28.42741307
N	27.61721848	29.36705380	28.43163028
C	28.76906396	28.65553182	28.57954265
H	31.94122586	28.14226189	28.55621691
H	25.67026004	26.89189236	29.08992465
H	24.49554247	28.76584767	28.71322373
H	25.34575789	30.20117507	28.30556969
N	21.15086880	26.28435919	28.89375568
C	22.44293189	26.76018063	28.85868538
O	22.69794307	27.92843564	29.06577656
N	23.45857153	25.86405706	28.57588555
C	23.20065270	24.60723824	28.40694239
N	24.19276569	23.76406827	28.05285266
C	21.86470889	24.05472098	28.54615254
C	20.87955459	24.93431710	28.78809130
H	25.12812455	24.10965457	28.06650954
H	24.07574768	22.78361193	28.18691110
H	21.67070705	22.99993320	28.45274023
H	19.85488024	24.62727509	28.91231485
H	30.29059334	30.04967190	28.17158836
H	20.42597840	26.93966534	29.10278270

29

S2S1T2 (6SeGua-Cyt in DNA)

N	30.38422801	29.16631558	28.13505128
C	31.31773822	28.14741964	28.23508481
N	30.87144896	27.02726748	28.62488563
C	29.50743986	27.25528551	28.84487736
C	28.47783941	26.43673824	29.12417646
Se	28.63632953	24.53680317	29.39881719
N	27.22328235	26.97668703	29.14855669
C	27.06590176	28.39346110	29.23600030
N	25.72029180	28.76092279	29.22849595
N	28.04195062	29.19651326	28.60486220
C	29.17388767	28.64108597	28.53676138
H	32.35141463	28.33483912	27.99071047
H	26.49491579	26.46998059	29.60906289
H	25.61888548	29.74887102	29.36521448
H	25.21734166	28.49433355	28.39792737
N	21.39784655	26.45242912	28.84637232
C	22.59729218	27.12559393	28.69452533
O	22.66797041	28.32646194	28.80868358
N	23.71530000	26.36763256	28.38245996

C	23.64677553	25.08356612	28.32300708
N	24.79194197	24.38265189	28.06257964
C	22.42041582	24.34076339	28.55909962
C	21.32652543	25.07723907	28.81399798
H	25.53264796	24.92846016	27.66779943
H	24.68827453	23.47530302	27.65725500
H	22.39419864	23.26391378	28.55645293
H	20.36233961	24.63624594	29.01163380
H	30.55012727	30.10993741	27.86308899
H	20.60107853	27.00572530	29.08253724

29
S1-MIN-U (6SeGua-Cyt in DNA)

N	29.93787238	29.39807478	28.46188692
C	30.68738775	28.25059110	28.51948042
N	29.96966686	27.18768902	28.57638351
C	28.67284076	27.64471916	28.56821513
C	27.43298292	26.92411111	28.48569494
Se	27.37452377	25.45500739	27.08063128
N	26.32901705	27.79582218	28.46513503
C	26.42596304	29.15650628	28.44476851
N	25.26082629	29.82335747	28.47710843
N	27.54302709	29.81708862	28.41368613
C	28.63273138	29.00413186	28.48975031
H	31.76367174	28.27510652	28.50394368
H	25.41779821	27.38683742	28.54653686
H	24.38298776	29.34504546	28.46323179
H	25.30155121	30.81531762	28.40588160
N	21.42788728	26.25828330	28.91491981
C	22.70935513	26.74701864	28.76805386
O	22.93509119	27.94051099	28.78223337
N	23.73575249	25.84471332	28.61077947
C	23.50219279	24.56749326	28.60093273
N	24.52552394	23.70641047	28.51931517
C	22.15999008	24.02082081	28.70925879
C	21.16666291	24.90602579	28.87640752
H	25.45984122	24.04671467	28.40563533
H	24.36029978	22.73155432	28.40750299
H	21.97534548	22.95957330	28.69756876
H	20.14035128	24.60089459	28.99925953
H	30.26990775	30.33287290	28.36842007
H	20.70446853	26.92885418	29.06887862

29
S2-MIN-U (6SeGua-Cyt in DNA)

N	29.97488745	29.29341355	28.47999743
C	30.81071550	28.21593176	28.69862315
N	30.18274782	27.12842920	28.92925850
C	28.85005568	27.46711062	28.84596733
C	27.68207164	26.68072950	29.00041186
Se	27.78629312	24.95061224	27.70007055
N	26.51857879	27.43447755	28.79613418
C	26.49675193	28.76964063	28.55027927
N	25.30085166	29.35295137	28.45794687
N	27.57405898	29.50413427	28.41163616
C	28.70826635	28.80902695	28.55383187
H	31.88230195	28.31838596	28.66242880
H	25.63446405	26.95611509	28.82628767
H	24.43593698	28.87409861	28.63466903
H	25.29287947	30.33074274	28.26983037
N	21.27909216	26.35217995	28.93218029
C	22.55201780	26.87237787	28.84827672
O	22.75497524	28.06329573	28.99283746
N	23.59320542	26.01380454	28.59001854

C	23.38413939	24.73541776	28.46732979
N	24.41628986	23.92124727	28.22430576
C	22.06452971	24.14628770	28.62302287
C	21.05270946	24.99485364	28.85334871
H	25.30479369	24.31860972	27.97040076
H	24.24671919	22.97479638	27.96436818
H	21.90867439	23.08208737	28.56793420
H	20.03965240	24.65692925	28.99726367
H	30.23698376	30.20390652	28.17116179
H	20.54229218	26.99772408	29.12527124

29
T1-MIN-U (6SeGua-Cyt in DNA)

N	29.95634358	29.23752057	28.49419015
C	30.75767794	28.13558476	28.68590274
N	30.10079011	27.05399518	28.87779417
C	28.77812578	27.42690933	28.79986508
C	27.58067047	26.65594247	28.91008240
Se	27.56200844	25.04977290	27.70557924
N	26.43069247	27.47964097	28.78032223
C	26.45565686	28.80873677	28.56676994
N	25.27008507	29.42991359	28.45937944
N	27.55474328	29.51647820	28.44031542
C	28.67219712	28.78432648	28.56314950
H	31.83198202	28.21027155	28.66214113
H	25.53294319	27.03129766	28.82533263
H	24.41637880	28.96687701	28.71426804
H	25.29743597	30.42596512	28.47784594
N	21.22858275	26.29612963	28.93804271
C	22.52204727	26.77369116	28.88454467
O	22.76198115	27.95864704	29.01397767
N	23.54089301	25.87185821	28.68680530
C	23.28960142	24.60041729	28.58665684
N	24.29361535	23.73811329	28.39789946
C	21.94345139	24.06171134	28.67352394
C	20.95603180	24.94826059	28.85577779
H	25.23344369	24.07981841	28.34060617
H	24.13415989	22.75719687	28.44036161
H	21.74961842	23.00441001	28.60974494
H	19.92647102	24.64671815	28.95286948
H	30.25432068	30.14500326	28.20287220
H	20.50505278	26.96196180	29.10678964

29
T2-MIN-U (6SeGua-Cyt in DNA)

N	29.95402710	29.24781559	28.46333893
C	30.73955073	28.12762563	28.61844846
N	30.05627339	27.06154611	28.80116707
C	28.74313479	27.47453296	28.76378768
C	27.53848559	26.71260645	28.81330698
Se	27.63336025	25.02402025	27.65020235
N	26.40663261	27.53848977	28.65035698
C	26.45439279	28.88381163	28.46452846
N	25.26869231	29.51234536	28.32942655
N	27.55036109	29.58054642	28.38995707
C	28.66357604	28.82031027	28.52966842
H	31.81450230	28.18274681	28.57934240
H	25.50447232	27.10383871	28.70096729
H	24.42382271	29.05961245	28.62152054
H	25.31509772	30.50514691	28.40941073
N	21.25517265	26.25585952	28.91529456
C	22.55591158	26.71176557	28.83742527
O	22.82106120	27.88903269	28.96483831
N	23.55783677	25.78802227	28.61821165

C	23.28068731	24.52530577	28.51563300
N	24.26681496	23.63755009	28.30119854
C	21.92729890	24.01054083	28.61861404
C	20.95964010	24.91362528	28.82712681
H	25.21807695	23.93522118	28.34493614
H	24.08529000	22.66178756	28.37446470
H	21.71106101	22.95820495	28.54815736
H	19.92735921	24.62851094	28.94182019
H	30.25843082	30.16384286	28.22330241
H	20.54313161	26.93151154	29.10272549

29
S2S1-U (6SeGua-Cyt in DNA)

N	30.08830056	29.32637312	28.45469105
C	30.96736880	28.27760465	28.65158907
N	30.38712406	27.15462117	28.82824107
C	29.03580420	27.43723665	28.75088050
C	27.89477913	26.62112936	28.91904067
Se	28.00729928	24.88429709	27.55377396
N	26.70800615	27.33758678	28.76155056
C	26.63671381	28.67844951	28.53542924
N	25.42496168	29.22448973	28.44769810
N	27.68877824	29.44839768	28.41307457
C	28.84501881	28.78952524	28.52351959
H	32.03419646	28.42824630	28.63781931
H	25.83987792	26.82986034	28.78204837
H	24.56886397	28.72395490	28.61267305
H	25.39219307	30.20580376	28.28002356
N	21.43465023	26.24249387	28.83231255
C	22.70095309	26.78145697	28.75087647
O	22.88755565	27.97065924	28.93481027
N	23.74980446	25.94918676	28.45429828
C	23.55727030	24.67029860	28.29062475
N	24.58554429	23.88943913	27.96622407
C	22.24943808	24.05654186	28.46425327
C	21.22892627	24.88336541	28.73404738
H	25.49330470	24.28375699	27.77512951
H	24.45137170	22.91507539	27.81501141
H	22.10911214	22.99142317	28.38904688
H	20.22303513	24.52833051	28.89040811
H	30.30666913	30.28705741	28.30467944
H	20.69315882	26.86534092	29.07342922

29
T2T1S0-U (6SeGua-Cyt in DNA)

N	29.79929387	29.30677350	28.60235950
C	30.51825109	28.12282323	28.65773670
N	29.78913651	27.08023732	28.67091219
C	28.48441253	27.55638786	28.64035546
C	27.27634698	26.86889890	28.53172532
Se	27.42288448	25.08906577	26.53319534
N	26.21945786	27.74914222	28.51831524
C	26.29899987	29.10307849	28.53468967
N	25.14724343	29.79103351	28.49903292
N	27.43609646	29.75380963	28.55561145
C	28.49263372	28.94559655	28.60571234
H	31.59559585	28.12877116	28.67062160
H	25.30080246	27.33782234	28.48672824
H	24.27494823	29.32966580	28.68232642
H	25.21672467	30.77017126	28.67238942
N	21.30896253	26.33770355	28.87629318
C	22.55419026	26.92425203	28.83410496
O	22.69568816	28.12719562	28.94594797
N	23.64700857	26.11103315	28.66011065

C	23.52372467	24.81824835	28.61349239
N	24.61994448	24.05173606	28.55384680
C	22.22597533	24.16591921	28.67723583
C	21.15923673	24.96743587	28.81580100
H	25.51045266	24.48755226	28.40062900
H	24.53814138	23.10315348	28.25898065
H	22.12773498	23.09327208	28.65893935
H	20.15795111	24.57841069	28.90410564
H	30.15531424	30.23770366	28.60281279
H	20.52625822	26.94036835	29.01858879

29

T1S0-U (6SeGua-Cyt in DNA)

N	29.79091471	29.26427729	28.61643776
C	30.46683202	28.07649329	28.75920643
N	29.69333151	27.05605465	28.76377151
C	28.42190987	27.57848803	28.63309706
C	27.09853970	26.92976300	28.59160793
Se	27.21455120	26.30112917	26.66019039
N	26.10709314	27.92261473	28.80522480
C	26.28678198	29.26055731	28.61974743
N	25.16884060	30.00378015	28.61535004
N	27.44132651	29.83681262	28.47802144
C	28.46967780	28.94311945	28.54644690
H	31.54078257	28.04139601	28.83052678
H	25.16766019	27.58234906	28.83097721
H	24.26768614	29.59209614	28.74542669
H	25.27312507	30.99189297	28.55757387
N	21.33865153	26.29999909	28.86572288
C	22.57433167	26.90641110	28.84423616
O	22.70485516	28.10733337	28.96386312
N	23.67935831	26.10117656	28.69001252
C	23.57394322	24.81506022	28.60944875
N	24.69695849	24.07920544	28.47406987
C	22.28734794	24.14073894	28.64718199
C	21.20832173	24.92894859	28.78401901
H	25.57648445	24.52696945	28.64250040
H	24.65792765	23.08886923	28.57199158
H	22.19969327	23.06792807	28.60009822
H	20.21079426	24.52638947	28.85351028
H	30.16649536	30.18680440	28.63581528
H	20.54325521	26.89031348	28.98966480

29

S1-MIN-D (6SeGua-Cyt in DNA)

N	29.95165809	29.16717891	28.30341339
C	30.70307938	28.03168337	28.46714455
N	29.99002224	27.00263786	28.75560828
C	28.70082075	27.47205040	28.79756642
C	27.46990274	26.79512406	29.08466611
Se	27.52486168	25.45929699	30.59353363
N	26.36843003	27.67639923	29.06861225
C	26.46251089	28.99920546	28.73616550
N	25.29860889	29.66607775	28.67379214
N	27.56974076	29.62506199	28.48480139
C	28.65684995	28.80661186	28.52617649
H	31.77243587	28.03574835	28.33842131
H	25.45457523	27.26324645	29.01793298
H	24.42820186	29.25224473	28.94123812
H	25.35309279	30.64584468	28.50578129
N	21.32479045	26.36206074	28.91366326
C	22.57072697	26.95100979	28.92213557
O	22.71597298	28.13647838	29.13698233
N	23.66659119	26.14769271	28.68041717

C	23.53769011	24.86803505	28.52720411
N	24.62773908	24.12034325	28.25316577
C	22.24736878	24.20428348	28.60920153
C	21.18022201	24.99448079	28.80387491
H	25.52639216	24.54379854	28.37729595
H	24.58782137	23.13899300	28.42655900
H	22.15087903	23.13465630	28.53084860
H	20.18131620	24.59968968	28.89627030
H	30.27892284	30.09046807	28.13448649
H	20.53768132	26.94916399	29.09790464

29
S2-MIN-D (6SeGua-Cyt in DNA)

N	30.03070754	29.34224634	28.32813706
C	30.78706396	28.20902090	28.55258150
N	30.10515119	27.22092555	28.98727786
C	28.80991211	27.69256432	29.06427904
C	27.61063208	27.06702779	29.47069320
Se	27.72208017	26.28127148	31.53054721
N	26.51627996	27.91824282	29.32910309
C	26.56479735	29.19317325	28.88017178
N	25.40485695	29.85944204	28.81767927
N	27.67340243	29.79589383	28.53000162
C	28.75206168	29.01179635	28.64236157
H	31.84665219	28.19811138	28.35572237
H	25.60833277	27.51385201	29.47014693
H	24.52063347	29.40074739	28.93998440
H	25.43095508	30.75953198	28.38776990
N	21.57827007	26.40621822	28.97346552
C	22.78054240	27.07905051	28.98972320
O	22.83968472	28.28378903	29.13654692
N	23.93315736	26.34229112	28.84125903
C	23.89418296	25.05701957	28.68665617
N	25.05046222	24.38191442	28.51138738
C	22.64494652	24.31715739	28.66343510
C	21.52276551	25.03744483	28.82265016
H	25.89972872	24.86920686	28.72270360
H	25.05934572	23.40086324	28.69262536
H	22.61913164	23.24624828	28.55332301
H	20.54623573	24.58149327	28.85467287
H	30.35269745	30.22361314	27.98782666
H	20.75001235	26.94041016	29.12290137

29
T1-MIN-D (6SeGua-Cyt in DNA)

N	29.94088609	29.14055621	28.29621135
C	30.69199964	27.99715657	28.41866852
N	29.98008253	26.95993979	28.67341288
C	28.68716962	27.42837476	28.74036457
C	27.45791072	26.73201882	29.00427057
Se	27.55648885	25.44112273	30.60293399
N	26.36430531	27.61271394	28.93844317
C	26.45691989	28.95619183	28.71705044
N	25.29497303	29.62799306	28.69834066
N	27.56787960	29.59469656	28.51008874
C	28.64806727	28.77298891	28.51771726
H	31.76066623	28.00431144	28.28323027
H	25.44274271	27.21597573	28.94878606
H	24.41975927	29.20098144	28.92911623
H	25.35177412	30.61721493	28.60030187
N	21.28277250	26.39866318	28.91680661
C	22.51990349	26.99780212	28.90637681
O	22.66258696	28.18572243	29.11009230
N	23.62054223	26.20015262	28.65553214

C	23.50538200	24.91905418	28.51215766
N	24.59782927	24.18402045	28.22380788
C	22.22351824	24.24085076	28.62732513
C	21.15007359	25.02486357	28.83053800
H	25.49700569	24.62017647	28.27318772
H	24.57635952	23.19703906	28.35810907
H	22.13607524	23.16914911	28.56928756
H	20.15659977	24.62287298	28.94516386
H	30.25806785	30.07348290	28.15278812
H	20.49069304	26.97970177	29.09628870

29

T2-MIN-D (6SeGua-Cyt in DNA)

N	29.96125722	29.14464957	28.28708398
C	30.73719361	28.01374327	28.40067568
N	30.05605584	26.96199542	28.66594230
C	28.75170859	27.39666935	28.75722011
C	27.54290303	26.67154487	28.98836308
Se	27.57293524	25.42733767	30.60938163
N	26.42911970	27.54411945	28.95397470
C	26.48458253	28.87723905	28.72008655
N	25.31859487	29.53779351	28.70157689
N	27.58988473	29.53926285	28.50579404
C	28.68048313	28.75020085	28.52214838
H	31.80398444	28.04489506	28.25208233
H	25.51625262	27.13154529	29.01001076
H	24.43969610	29.10449989	28.90859568
H	25.36510182	30.52534850	28.58338012
N	21.27909161	26.41713728	28.90896729
C	22.50927148	27.02747324	28.88393294
O	22.64521841	28.21592413	29.09006849
N	23.61440233	26.24153646	28.61311649
C	23.50982793	24.96014234	28.47827426
N	24.60608005	24.23484273	28.16264321
C	22.23820580	24.26825341	28.61678604
C	21.15923253	25.04103830	28.83209545
H	25.49493500	24.68841254	28.23293909
H	24.60320409	23.25605168	28.35123599
H	22.16290738	23.19527979	28.56671066
H	20.17175407	24.63003672	28.96450175
H	30.25623027	30.08610708	28.14953615
H	20.48334187	26.98987672	29.09760251

29

S2S1-D (6SeGua-Cyt in DNA)

N	30.12008731	29.21463852	28.55448797
C	30.98710069	28.13197721	28.48488612
N	30.39770147	27.00356165	28.48571490
C	29.04849414	27.30130938	28.59253150
C	27.89719850	26.49697055	28.47053764
Se	27.99178044	24.89007914	30.00129656
N	26.72028213	27.25697738	28.56967636
C	26.66964562	28.59994676	28.67885552
N	25.47970896	29.18326893	28.80168485
N	27.74016806	29.36974539	28.67779207
C	28.87593806	28.68876459	28.63360104
H	32.05384670	28.27541411	28.42584053
H	25.84427657	26.76289560	28.59334430
H	24.60288899	28.69547817	28.74950507
H	25.47974660	30.17838990	28.84862784
N	21.44183478	26.23853473	28.69885494
C	22.69984823	26.79346615	28.67838899
O	22.85964342	27.99837712	28.71831261
N	23.78513632	25.95122309	28.62178589

C	23.62792136	24.66357510	28.59568357
N	24.72130369	23.87581268	28.58878900
C	22.31298108	24.04550298	28.56229665
C	21.25878906	24.87315252	28.62816032
H	25.55470104	24.28343656	28.98769798
H	24.58803434	22.91066523	28.80628299
H	22.18973696	22.97616024	28.53571207
H	20.24347416	24.51198503	28.65885928
H	30.34990278	30.18405913	28.58401680
H	20.66841749	26.86749190	28.74729209

29

T2T1S0-D (6SeGua-Cyt in DNA)

N	29.79515200	29.28330484	28.51810551
C	30.49694997	28.10086422	28.69476617
N	29.74950374	27.08385011	28.85978537
C	28.45253918	27.57809597	28.79719193
C	27.22636124	26.92653045	28.92474835
Se	27.17931968	25.19033492	30.97672988
N	26.19114491	27.81971055	28.78569117
C	26.29521402	29.15567065	28.58413069
N	25.15265341	29.85168236	28.46668763
N	27.44325920	29.77618619	28.47810224
C	28.48435739	28.95236583	28.58464510
H	31.57417551	28.08760553	28.67347191
H	25.26438653	27.42609465	28.80914801
H	24.27791435	29.43825613	28.73184219
H	25.23878771	30.84467652	28.48420233
N	21.33306285	26.38053270	28.93576204
C	22.55102332	27.01706437	28.88626167
O	22.65777447	28.21604906	29.05840680
N	23.66571069	26.25262722	28.63886501
C	23.59431669	24.96218508	28.52826511
N	24.71538976	24.26233679	28.27224670
C	22.33067221	24.25264983	28.64532768
C	21.23838736	25.00595022	28.85070368
H	25.58901979	24.73313040	28.42742231
H	24.72030301	23.29016267	28.49510546
H	22.27218495	23.17866648	28.58683218
H	20.25680507	24.57669521	28.97144849
H	30.16805243	30.19667030	28.37587600
H	20.53415725	26.94300070	29.13998900
